# Single-cell transcriptomics and RNAi screening define a hierarchical program of planarian eye regeneration

**DOI:** 10.1016/j.celrep.2026.117245

**Published:** 2026-04-09

**Authors:** M. Lucila Scimone, Bryanna I. Canales, Patrick Aoude, Kutay D. Atabay, Cyrille W. Teforlack, Peter W. Reddien

**Affiliations:** 1 Howard Hughes Medical Institute, Massachusetts Institute of Technology, Cambridge, MA 02139, USA; 2 Whitehead Institute for Biomedical Research, Cambridge, MA 02142, USA; 3 Department of Biology, Massachusetts Institute of Technology, Cambridge, MA 02139, USA; 4 Department of Computational and Systems Biology, Massachusetts Institute of Technology, Cambridge, MA 02139, USA; 5 Duke University, Durham, NC 27708, USA; 6 Lead contact

## Abstract

The evolution and development of eyes are fundamental problems in biology, and numerous genetic and age-related degenerative eye diseases are still poorly understood. Planarians are flatworms that are able to fully regenerate functional eyes following injury, presenting a powerful model to study essential attributes of eye biology and regeneration. We performed single-cell eye transcriptomic analyses and large-scale RNA interference screening to define a hierarchical sequence of steps in eye regeneration and the genes that control each step in this process: from progenitor specification to differentiation into mature photoreceptors and melanin-pigmented optic cup cells, rhabdomere and dorsal projection formation in photoreceptors, eye morphogenesis (a self-organizing process where eyes trap progenitors and promote their differentiation), and interactions with the surrounding extracellular environment to produce a transparent region for light transmission. This hierarchical program defines roles for many conserved genes and establishes a framework for the regeneration of an entire organ.

## In brief

Some organisms have the capacity to regenerate missing organs *de novo*. Scimone et al. systematically identify the genes that control a sequence of organ-regeneration steps from differentiation of progenitors to the emergence of final architecture for the case of the planarian eye.

## Graphical Abstract

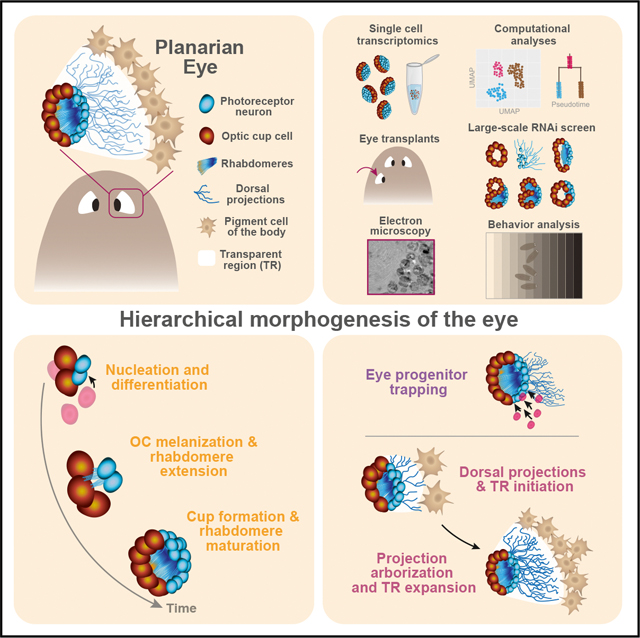

## INTRODUCTION

Some organisms have the ability to regenerate missing organs *de novo*. Dissection of the molecular and cellular processes involved in adult organ regeneration, however, has been limited by technical constraints. Planarians are flatworms with the ability to fully regenerate adult organs, and any gene can be inhibited with RNA interference (RNAi), giving planarians the potential to be one of the rare venues where large-scale gene perturbation studies can be performed in an adult context for systematic study of organ regeneration mechanisms.^1^ *De novo* organ formation in planarian regeneration involves fate specification in adult pluripotent, mesenchymal stem cells (neoblasts), the migratory targeting of progenitors to particular locations guided by extrinsic cues, the nucleation and differentiation of progenitors into nascent organs, the organization of regenerating cells into the correct form, and the interaction of regenerating cells with surrounding adult tissues for integration with anatomy. The identity and roles of genes that control these steps of organ regeneration are poorly understood. Among adult planarian tissues, eyes are attractive targets for uncovering molecular and cellular principles of organ regeneration. Planarian eyes are discrete, visible, dispensable for viability, and are accessible for extensive manipulation, including full or partial resection and even transplantation.^2^ Planarian eye regeneration can be studied during head regeneration or following resection. Genes with roles in development or the regeneration of other tissues, therefore, can still be studied in eye regeneration by inhibiting the gene and resecting the eyes. These attributes open the path to systematic molecular and cellular investigation of eye regeneration mechanisms in this model system. Prior studies of planarian eye regeneration have uncovered stem cell fate-specification mechanisms, properties underlying progenitor migratory targeting and self-organization, and the existence of adult guidepost cells impacting axon regeneration and guidance.^3–9^ However, the molecular mechanisms of most steps of planarian eye regeneration remain poorly understood. Genes associated with these steps might reveal broadly important roles in animal eye biology and disease.

**Figure 1.**
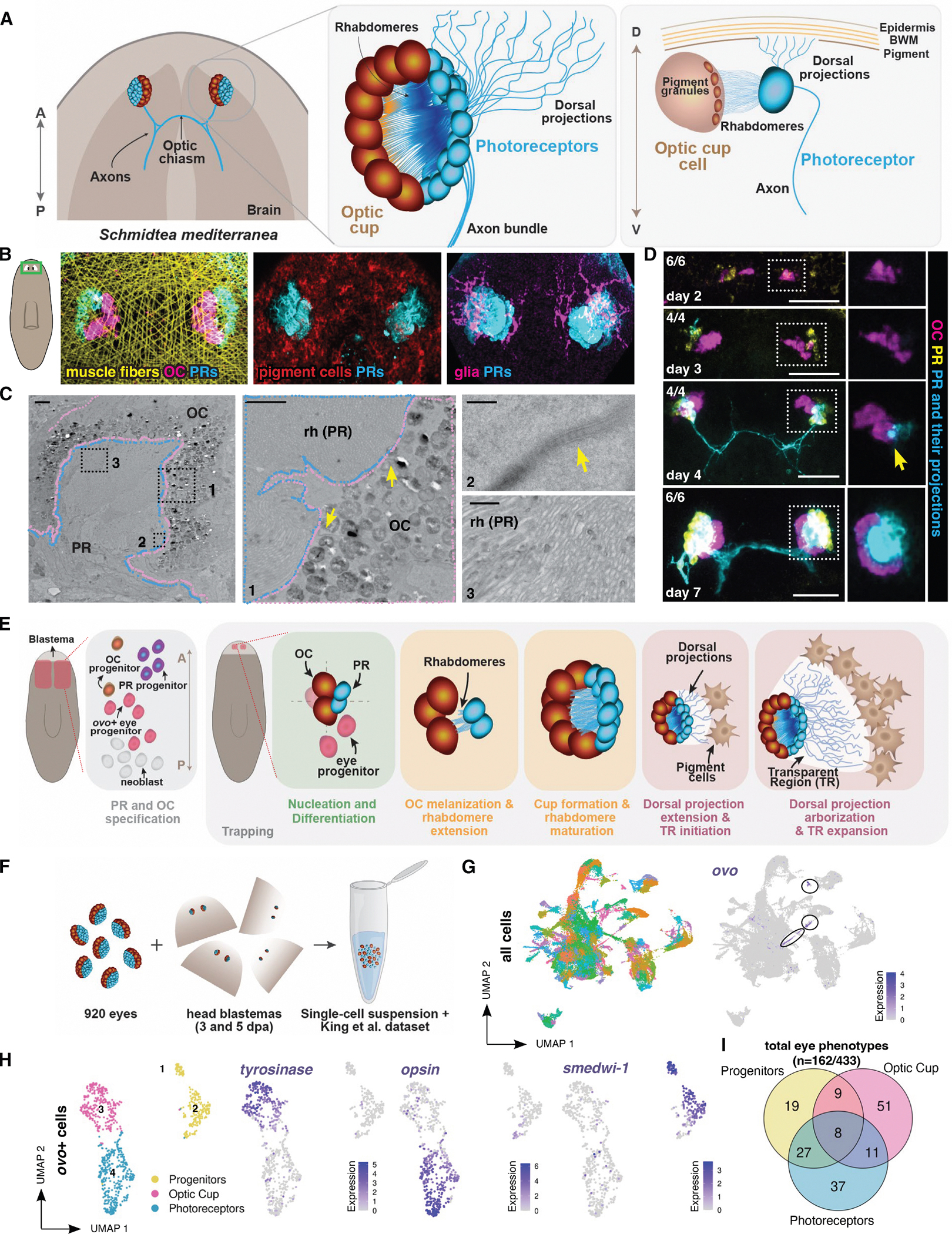
Stages in planarian eye regeneration and single-cell eye transcriptomics (A) Schematics of the planarian eye. (B) FISH and immunostaining showing neighboring cell types of the planarian eye in a wild-type uninjured animal. Cartoon shows the region of images displayed. (C) TEM showing distinct features of OC and PR cells in a wild-type uninjured animal. Dotted boxes 1, 2, and 3 are shown in the middle and right. Yellow arrows mark adherens junctions. Rh, rhabdomeres. </p/>(D) FISH and immunostaining showing an eye regeneration time course following head amputation in wild-type animals. Yellow arrow points to initial rhabdomere formation. Dotted white box with only OC and Arrestin labeling is shown on the right. (E) Schematics of eye regeneration stages. (F) Schematics of single-cell experimental design. (G) UMAP plots showing 133 cell clusters and *ovo* expression in 3/133 clusters. (H) UMAP plots showing subclustering of *ovo*+ cells, and the expression of the OC marker *tyrosinase*, the PR marker *opsin*, and the progenitor marker *smedwi-1.* (I) Venn diagram shows the number of phenotypes following RNAi and their distribution based on expression in the different eye cell types. Scale bars: 2 mm (left), 500 nm (1) and (3), and 100 nm (2) in (C) and 50 μm in (D).

The planarian eye is composed of two major cell types: pigmented optic cup (OC) epithelial cells and light-sensing photoreceptors (PRs) (Figure 1A). The OC is a specialized cup-shaped structure with melanin pigmentation that shields light from photosensitive PRs. Several similarities between planarian OC cells and the vertebrate retinal pigment epithelium (RPE) have previously been noted.^4^ In both cases, these cells contain melanin granules that are believed to offer protection from oxidative damage.^10,11^ In addition, numerous metabolite and solute transporters are expressed in the planarian OC and the vertebrate RPE, consistent with their role as a transport epithelium supporting PR biology.^12^

Planarian PRs have three distinct types of projections involving membrane specialization (Figure 1A). Their apical membranes form rhabdomeres—microvilli-like structures—that project into the OC. PRs also extend stereotypical dorsolateral projections (in short, dorsal projections) from the eye to a location just ventral to the dorsal body wall muscle (BWM) fiber network. On the basal side, PRs project axons that bundle together and connect to the brain ipsilaterally and contralaterally.^13,14^ We previously showed that the navigation of regenerating PR axons is facilitated by extrinsic cues that include muscle and neuronal guidepost-like cells found along the axonal tracts at key positions.^7^ This strategy allows for the precise regeneration of the visual circuitry in the adult organism.

We manually isolated 920 eyes and analyzed the transcriptomes of individual eye cells and their progenitors. We then performed an RNAi screen of 435 eye-expressed genes, with a focus on genes encoding adhesion molecules, cytoskeleton components, membrane-bound receptors, transcriptional regulators, and uncharacterized proteins, to determine their potential roles in eye regeneration steps. We identified genes with essential roles at each stage of eye regeneration, including the specification and differentiation of PRs and OC cells, the capture of progenitor cells into existing eyes for growth and maintenance, the morphogenesis of the OC, the OC cell pigmentation, the generation of PR projections, including rhabdomeres and dorsal projections, and the occlusion of body pigment from PR dorsal projections to allow light passage to the eye. These genes and associated cellular steps define a hierarchical path from stem cells to the regeneration of a mature organ.

## RESULTS

### Characterization of planarian eye regeneration stages

Planarian eyes are just ventral to BWM fibers (Figures 1A and 1B and Video S1). Body pigment cells surround the eye but are not in direct contact with PR cell bodies^15^ (Figure 1B and Video S2). Glia are interspersed with PRs^16,17^ (Figure 1B and Video S3). The tight organization of rhabdomeres projecting into the OC is apparent using transmission electron microscopy (TEM)^18^ (Figure 1C). OC cells display adherens junctions on their apical side (Figure 1C), generating a tight barrier, similar to the vertebrate RPE.^19,20^ Planarian OC cells, like RPE cells, are polarized with melanin granules concentrated on the apical side in close proximity to the PR rhabdomeres (Figure 1C).

Planarian eye regeneration starts with the specification of eye progenitors from pluripotent stem cells called neoblasts. This involves the expression of eye-associated transcription factors (TFs) in a small subset of neoblasts to produce eye-specialized neoblasts.^3,4^ Eye-specialized neoblasts divide to generate post-mitotic progenitors (pmPs) that can migrate and differentiate into eye cells at precise locations, defined by adult positional information.^6,8^ Neoblasts do not rapidly migrate under homeostatic conditions; however, their descendant cells (pmPs) migrate and can incorporate into tissues during both normal tissue maintenance and regeneration.^21–28^ We sought to further temporally define the steps of eye regeneration to frame the study of molecular mechanisms around that sequence of events (Figures 1D, 1E, and S1A). Eye progenitors expressing the eye-specific TF *ovo* migrate to bilaterally symmetric locations called the target zone (where eye progenitors nucleate and self-organize into eyes) between 1 and 2 days following head amputation.^4,6^ Differentiation of eye progenitors into mature OC cells (*catalase-1*+ and *tyrosinase*+) or PRs (Arrestin+) was first apparent 2 days post-amputation (dpa, Figure 1D). At 3 dpa, an increased number of differentiated cells was present, with more OC cells than PRs (Figures 1D and S1B), suggesting faster differentiation of OC cells than PRs. At this time point, as previously shown,^7^ pioneer axons were first observed, with the optic chiasma forming between 3 and 4 dpa (Figure 1D). Between 4 and 5 dpa, the OC cell number plateaued (Figures 1D, S1A, and S1B); the OC remained generally disorganized, with the cup shape not fully formed (Figure 1D). Also around this time, the initial formation of rhabdomeres was observed in tight association with OC cells (Arrestin+ dense circle, Figure 1D). By 5 dpa, rhabdomere elongation inside the OC shape was apparent (Figure S1A). After 5 dpa, the incorporation of PRs into the eye continued, with numbers reaching a steady state plateau at approximately 10 dpa (Figure S1B). Between 7 and 10 dpa, PR dorsal projections became visible, reaching an elaborate arborization state by 15 dpa (Figure S1A).

Similar stages in eye regeneration were observed following resection of a single eye (Figures S1C and S1D^29^). This injury results in eye regeneration without production of progenitors above the basal rate because the small nature of the injury fails to elicit the sustained, elevated proliferation associated with a missing tissue response.^5^ In this context, the eye cell type that first appeared was variable (i.e., either OC cells or PRs were first observed; Figures S1C and S1D), presumably associated with the pre-existence of progenitors at different maturation stages at the time of injury. Additionally, OC structure and rhabdomeres appeared slightly earlier than in the head regeneration context, by day 3 post-resection (Figure S1C). These observations indicate the following order of events for eye regeneration, used below for framing gene function investigation: (1) PR and OC specification; (2) nucleation and differentiation; (3) eye morphogenesis, including OC melanization, cup formation, and rhabdomere extension; and (4) dorsal projection extension and arborization and transparent region (TR) formation (Figure 1E).

### single-cell RNA-sequencing of planarian eyes

To identify genes associated with eye regeneration stages, we performed single-cell RNA sequencing (scRNA-seq) (10×) from isolated eyes and from regenerating head blastemas at 3 and 5 dpa, which should enrich for eye progenitors (Figures 1F, S1E, and S1F). Eye regeneration involves continuous production and incorporation of progenitors during growth through this time window. We isolated 920 eyes from wild-type animals and dissociated them in a solution of trypsin and papain to preserve PR projections (see STAR Methods). To further increase eye-progenitor numbers, we combined this dataset with a previously published neoblast and pmP dataset from 3 dpa.^30^ Using Seurat, we clustered all cells and analyzed the expression of the eye-specific TF *ovo* (Figures 1G and S1G–S1I). From 133 total clusters, three clusters were composed of *ovo*+ cells (Figure 1G, Table S1). Cells in one of the three *ovo*+ clusters were *tyrosinase*+ (an OC cell marker), cells in another cluster expressed *opsin* (a PR marker), and cells in the third cluster expressed high levels of the neoblast marker *smedwi-1,* indicating that it was an eye progenitor-enriched cluster (Figure S1H). We isolated all *ovo*+ cells (see STAR Methods), subclustered them, and performed differential gene expression analysis to identify genes with enriched expression in OC cells, PRs, and progenitors, as well as genes expressed in all eye cells (Figures 1H and S1I and Table S2).

A total of 10,510 genes were expressed in eye cells in the scRNA-seq data (Figure S1J). Using RNAi, we assessed the function of 427 genes that either had enriched expression in one of the *ovo*+ clusters or that were expressed in multiple eye cell clusters (Tables S2 and S3). We also included, regardless of the expression of these genes in our dataset, a few additional genes that had previously been shown to affect eye regeneration (for a total of 435 genes, Figure S1J). Following six double-stranded RNA (dsRNA) feedings over 21 days, animals were decapitated, and eye regeneration was assessed at 7 dpa in regenerating head blastemas (Figure S1K). Additionally, in head fragments, we performed unilateral eye resection on the day of decapitation (0 dpa) (Figure S1K). In this injury context, head fragments rescale the positional information in order to properly regenerate,^6^ and the regenerating eye must trap incoming progenitors to maintain a two-eye configuration despite positional information shifting eye-progenitor targeting more anteriorly (see text below). For essential genes for which this RNAi protocol caused animal lysis, we repeated gene inhibition using only one or two dsRNA feedings over 7 days to assess any requirement in eye regeneration (Table S3). From all genes screened, 191 had RNAi phenotypes affecting regeneration and/or animal homeostasis, and 164 out of 191 genes displayed an eye phenotype after RNAi (Figure 1I and Table S3). These phenotype classes are dissected, assigning genes to roles in the stages of eye regeneration, below. This represents by far the largest collection of genes with functional requirements defined for the regeneration stages of any organ in a regeneration model system. We validated the expression of a subset of the genes selected for RNAi screening by fluorescence *in situ* hybridization (FISH) to determine the accuracy of the 10× scRNA-seq dataset (Figure S1L).

### Progenitor specification and differentiation: Differentiation trajectories for eye progenitors

**Figure 2.**
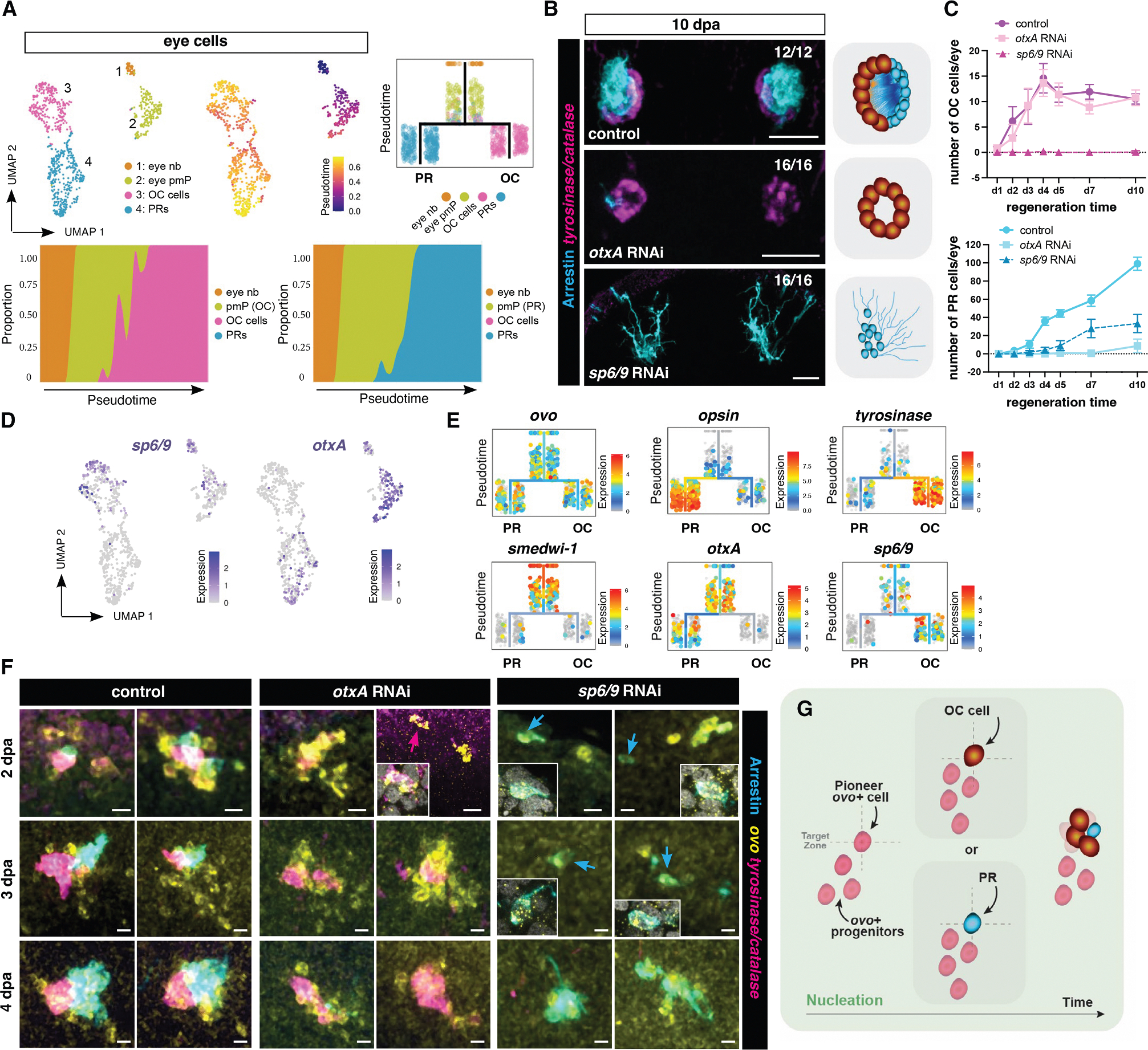
Pseudotime analyses and single eye progenitor differentiation (A) UMAP plot showing *ovo*+ cell subclustering (top left) and pseudotime analysis (top middle and bottom). URD trajectory of *ovo*+ clusters (top right). (B) FISH and immunostaining show eye regeneration phenotypes following decapitation of RNAi animals. (C) Graphs show OC and PR numbers (mean ± SD) during eye regeneration following decapitation. (D) UMAP plots showing *sp6/9* and *otxA* expression in *ovo*+ cells. (E) URD trajectory analyses show eye gene expression. (F) FISH and immunostaining show differentiation of a single *ovo*+ progenitor into either an OC or PR in *otxA* and *sp6/9* RNAi head blastemas. (G) Drawings summarize data observed. Scale bars: 50 μm in (B) and 10 μm in (F).

We defined four subclusters of *ovo*+ cells: OC cells (cluster 3, *tyrosinase*+), PRs (cluster 4, *opsin*+), and two expressing *smedwi-1* (progenitor clusters 1 and 2) (Figure 2A and Table S2). Analysis of cell cycle and neoblast markers in the progenitor clusters indicated that cluster 1 contained eye neoblasts and that cluster 2 contained eye pmPs (Figure S2A and Table S2). We used URD-based trajectory inference,^31^ rooted in cluster 1, to explore the cellular and molecular dynamics associated with the differentiated eye cell and progenitor populations (Figure 2A). We obtained two trajectories, one marking the PR trajectory and the other corresponding to the OC trajectory (Figure 2A).

Several genes encoding TFs required for regulating eye cell fates in planarians have been previously described.^3,4^ *sp6/9* is essential for OC differentiation, whereas *otxA* is required for PR differentiation (Figures 2B, 2C, and S2B). In the absence of OC cells (*sp6/9* RNAi), PRs were disorganized, with undetectable rhabdomere structure. In the absence of PRs (*otxA* RNAi), OC cells formed a closed spherical structure (Figures 2B and S2B). As expected, *sp6/9* was expressed in the OC trajectory, and *otxA* was expressed in the PR trajectory (Figures 2D and 2E). Interestingly, both uniform manifold approximation and projection (UMAP) plots and URD analysis showed that *sp6/9* was expressed in most cluster 1 cells (the neoblast cluster), whereas *otxA* was more abundantly expressed in cluster 2 (the pmP cluster) (Figures 2D and 2E). Moreover, we found some overlap in the expression of these two TFs (Figure S2C), suggesting that *sp6/9* is initially activated broadly in *ovo*+ eye-specialized neoblasts, becoming more specific in its expression to the OC fate later in the trajectory, whereas *otxA* was activated more specifically in PR progenitors. This observation might also explain previous results showing that *sp6/9* RNAi animals have a larger decrease in the numbers of *ovo*+ progenitors compared to the decrease observed in *otxA* RNAi animals.^4^ These trajectories enable identification of genes expressed at any stage from neoblast to eye differentiated cells (Table S2 as a resource, and see application of these trajectories below; genes of interest can also be queried at https://digieye.wi.mit.edu).

### Progenitor specification and differentiation: A single eye progenitor can differentiate into an eye cell

Many *ovo*+ progenitors simultaneously migrate to their target zone following decapitation between 1 and 2 dpa,^4^ where differentiation into fully mature OC cells and PRs occurs. The high numbers of *ovo*+ progenitors make it difficult to assess whether cell-cell contacts among *ovo*+ progenitors are required for the final differentiation of eye cells or if a single *ovo*+ progenitor has the potential to fully differentiate into either an OC cell or a PR autonomously. Using sp*6/9* and *otxA* RNAi animals, which have decreased *ovo*+ progenitors, we observed that single *ovo*+ progenitors could differentiate into PRs (Arrestin+) or OC cells (*tyrosinase/catalase*+) in the absence of other detectable *ovo*+ cells in their close vicinity (Figure 2F). This suggests that sustained contact between eye progenitors or between progenitors and differentiated eye cells is not strictly necessary as a nucleating event for the differentiation of OC cells and PRs. Instead, we suggest that the positional information environment of the target zone itself is sufficient to induce eye progenitor differentiation and nucleation, from even a single progenitor (Figure 2G).

### Progenitor specification and differentiation: Genes required for the differentiation of eye progenitors

**Figure 3.**
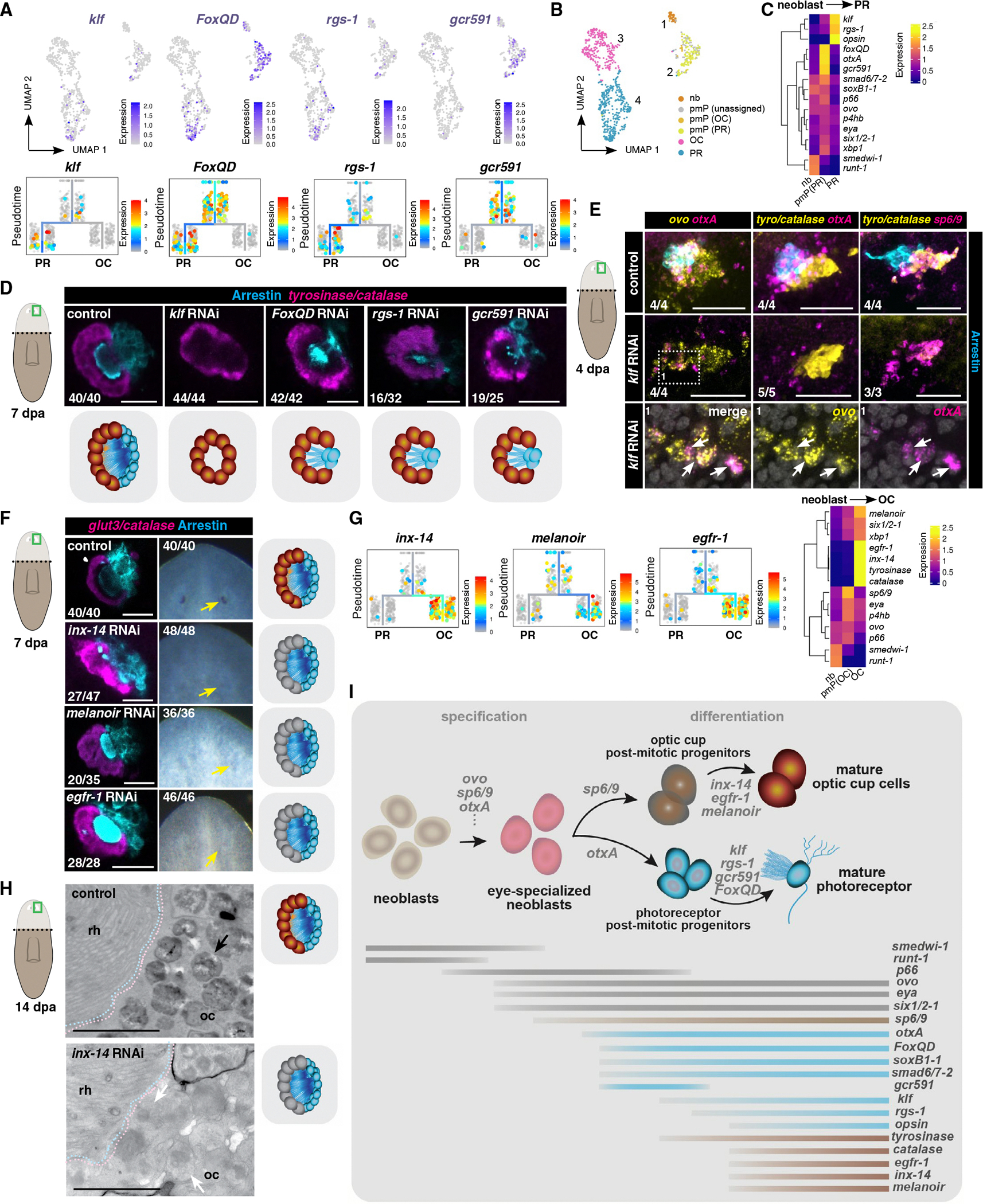
Genes involved in PR differentiation and melanin formation in OC cells (A) UMAP plots (top) and URD trajectory analyses (bottom) of genes required for PR differentiation. (B) UMAP plot shows the distribution of cells along the PR and OC differentiation path. (C) Heatmap shows gene expression along the PR differentiation trajectory. (D) FISH, immunostainings, and illustrations show PR regeneration defects following RNAi. (E) FISH and immunostainings show PR progenitors (*otxA*+ *ovo*+, white arrows) in a *klf* RNAi animal lacking mature PRs. (F) FISH, immunostainings, and illustrations show melanin regeneration defects following RNAi. Yellow arrows show melanin absence in OC cells in live RNAi animals. (G) URD trajectory analyses of genes required for melanin formation (left), and heatmap showing gene expression along the OC differentiation trajectory (right). (H) TEM shows lack of melanin pigment (white arrows) in OC cells of an *inx-14* RNAi animal compared to control (black arrows). (I) Drawings summarize genes involved in eye specification and differentiation (top) and gene expression (bottom). Cartoons of regenerating animals show regions of images displayed. Scale bars: 50 μm in (D) and (F), 20 μm in (E), and 500 nm in (H).

Four genes in the RNAi screen were required for PR differentiation. Two encode TFs (*klf* and *FoxQD*) previously found to be expressed in PRs,^4^ and the other two genes (dd_27074 and dd_9381) encode signaling factors with homology to an RGS (regulator of G protein signaling) protein and a G protein-coupled receptor (GPCR), respectively (Figure S3A). RGS proteins are negative regulators of G protein signaling, accelerating GTP hydrolysis by Ga subunits.^32^ We named dd_27074 *rgs-1* and dd_9381 *gcr591.* We assessed the expression of these and other genes known to be involved in eye progenitor specification and differentiation^3,4,23,33–35^ using UMAP plots and trajectory analyses. *gcr591* displayed the unusual characteristic of being expressed primarily in pmPs, consistent with a role in progenitor differentiation (Figures 3A and S3B). *FoxQD* was strongly expressed in PR progenitors and mature PRs, and *klf* and *rgs-1* were mostly expressed in differentiated PRs (Figure 3A). To better analyze the expression of these genes during progenitor differentiation, we split the eye pmPs (cluster 2) into PR pmPs (cells expressing *otxA* and *FoxQD* but not expressing *sp6/9*) and into OC pmPs (cells expressing *sp6/9*, *dlx*, tyrosinase, or *tph*^3,4,36^ but not *otxA* or *FoxQD*; Figures 3B and S3C and Table S1). Marker gene expression of PR pmPs was more correlated to PRs than to OCs, and OC pmP marker gene expression was more correlated to OCs than to PRs (Figure S3C). Small numbers of OC pmPs were observed. One possibility is that there is a rapid transition to differentiation for these cells or that they emerge later in the trajectory toward differentiation than previously appreciated. These possibilities could be of interest to study in the future to further dissect the mechanisms of differentiation in this trajectory. Trajectory analyses showed expression of the neoblast marker *smedwi-1* at early stages in pseudotime (in eye neoblasts), followed by the expression of general pmP markers associated with the transition toward differentiation^37^ (*p4hb* and *xbp1*), and finally, the expression of the differentiated PR marker *opsin* (Figure 3C). *runt-1*, a TF expressed in neoblasts and required for normal eye regeneration,^23,35^ was expressed in a largely overlapping manner with *smedwi-1* in the eye trajectory (Figures 3C, S3D, and S3E). *p66*, which encodes a member of the NuRD complex,^33^ as well as the newly described *gcr591* gene, reached their expression peak during the pmP stage, similar to the expression of general post-mitotic markers *xbp-1* and *p4hb* (Figures 3C, S3D, and S3E). The known eye TFs *six1/2–1*, *eya*, *ovo*, *otxA*, *soxB1–1*, *smad6/7–2*, and *FoxQD* were expressed in a pattern similar to one another, with high expression in pmPs and continued expression into fully differentiated PRs (Figures 3A–3C, S3D, and S3E). Finally, *klf* and *rgs-1* were strongly expressed in differentiated PRs (Figures 3A–3C and S3E), in a pattern similar to the PR marker *opsin*. The expression of some of these genes was not restricted to the eyes (Figure S3F and Table S1).

RNAi of some of these genes showed stronger effects than previously reported^23,33,35^ (Figures 3D and S3G and Table S3). *runt-1* RNAi animals formed tiny or no eyes at all (Figure S3G). *p66* RNAi animals did not form blastemas, consistent with a broad role for the NuRD complex in planarian regeneration^38^ (Figure S3G). *soxB1–1* and *smad6/7–2* RNAi animals lacked anterior PRs, as previously described^4,34^ (Figure S3G). *FoxQD* RNAi severely reduced PR numbers, whereas *klf* RNAi completely abolished PR differentiation (Figure 3D). Prior work with *klf* and *FoxQD* showed defects in eye morphogenesis,^4^ but current RNAi protocols enabled the uncovering of stronger phenotypes for these genes.

Inhibition of the genes *rgs-1* and *gcr591* delayed the differentiation of PRs, with eyes appearing normal by 28 dpa (Figures 3D and S3H). Inhibition of *gcr591* also mildly affected OC differentiation (Figure 3D). The complete absence of PRs following *klf* RNAi was observed even after 28 dpa (Figure S3H), indicating that PR differentiation in this context was not just delayed but completely blocked. We further confirmed the lack of PRs in *klf* RNAi eyes by TEM (Figure S3I). Notably, inhibition of *klf* did not affect the production of *otxA*+; *ovo*+ progenitors, even though Arrestin+ PRs were completely ablated (Figure 3E). These results with trajectory inference, together with FISH analyses of RNAi animals, suggest that *klf* is not required for the specification of PR progenitors, but for their capacity to differentiate.

### Progenitor specification and differentiation: Genes required for melanin formation in OC cells

Mammalian RPE and planarian OC cells both contain melanin. RPE melanin granules are thought to act as a protective barrier, absorbing excess light and mitigating damage to PR cells in the retina by neutralizing reactive oxygen species and reducing free radical damage.^10,39^ Age-related macular degeneration (AMD) is common and is thought to result from chronic oxidative stress damage within the RPE. AMD is associated with the decline of melanin.^39,40^ Understanding the mechanism of melanin formation and regeneration might provide better insights into this and other eye diseases.

We found three genes involved in the production of melanin in planarian OC cells (Figures 3F and S3J): an innexin gene (*inx-14*, dd_13805), a gene encoding an uncharacterized membrane protein containing Ig-like domains that we named *melanoir* (dd_10408, Figure S3K) and *egfr-1*. Innexins are components of gap junctions and hemichannels that allow ion and small molecule exchange between adjacent cells or with the extracellular milieu, respectively. Connexins possess this role in vertebrates, although these two protein groups are unrelated.^41^ The expression of several innexins has been previously reported in planarians, but specific roles during eye maturation or morphogenesis have not been described.^42–44^ Inhibition of *egfr-1* has previously been shown to affect planarian OC differentiation.^45^

UMAP plots, trajectory, and FISH analyses showed that *inx-14*, *melanoir*, and *egfr-1* were strongly expressed in mature OC cells (Figures 3G, S1L, S3L, and S3M). RNAi of these genes did not block the regeneration of *catalase*+ and *glut3*+ OC cells, indicating that they are not required for OC specification but specifically for melanin synthesis (Figure 3F). Inhibition of *inx-14*, but not the other two genes, affected the morphology of the regenerating OC (see below and Figure 3F). TEM of *inx-14* RNAi regenerating eyes also showed OC cells with immature melanosomes lacking melanin (Figure 3H). However, the expression of *tyrosinase* and *tph* (genes involved in melanin production) was not affected in any of the RNAi conditions (Figure S3N). Inhibition of *inx-14*, *melanoir*, and *egfr-1* affected negative phototaxis behavior (Figure S3O), pointing to the importance of melanization in the OC epithelium for light-response behavior.

Our screen revealed three genes that are involved in melanin formation in the planarian OC that have not been previously associated with melanogenesis in vertebrates, and it will be of interest to determine whether these genes also regulate melanogenesis in other organisms. Taken together, our results contribute to an understanding of the molecular regulation of specification and differentiation stages for PR and OC progenitors (Figure 3I). In summary, we show that a single progenitor can nucleate a new eye, determine the transcriptomes of maturing OC and PR progenitors, identify a collection of TFs and regulatory genes that specify fate and control differentiation of PRs, and define genes required for OC melanization.

### Regeneration of form: Genes involved in the formation of rhabdomeres and OC morphology

**Figure 4.**
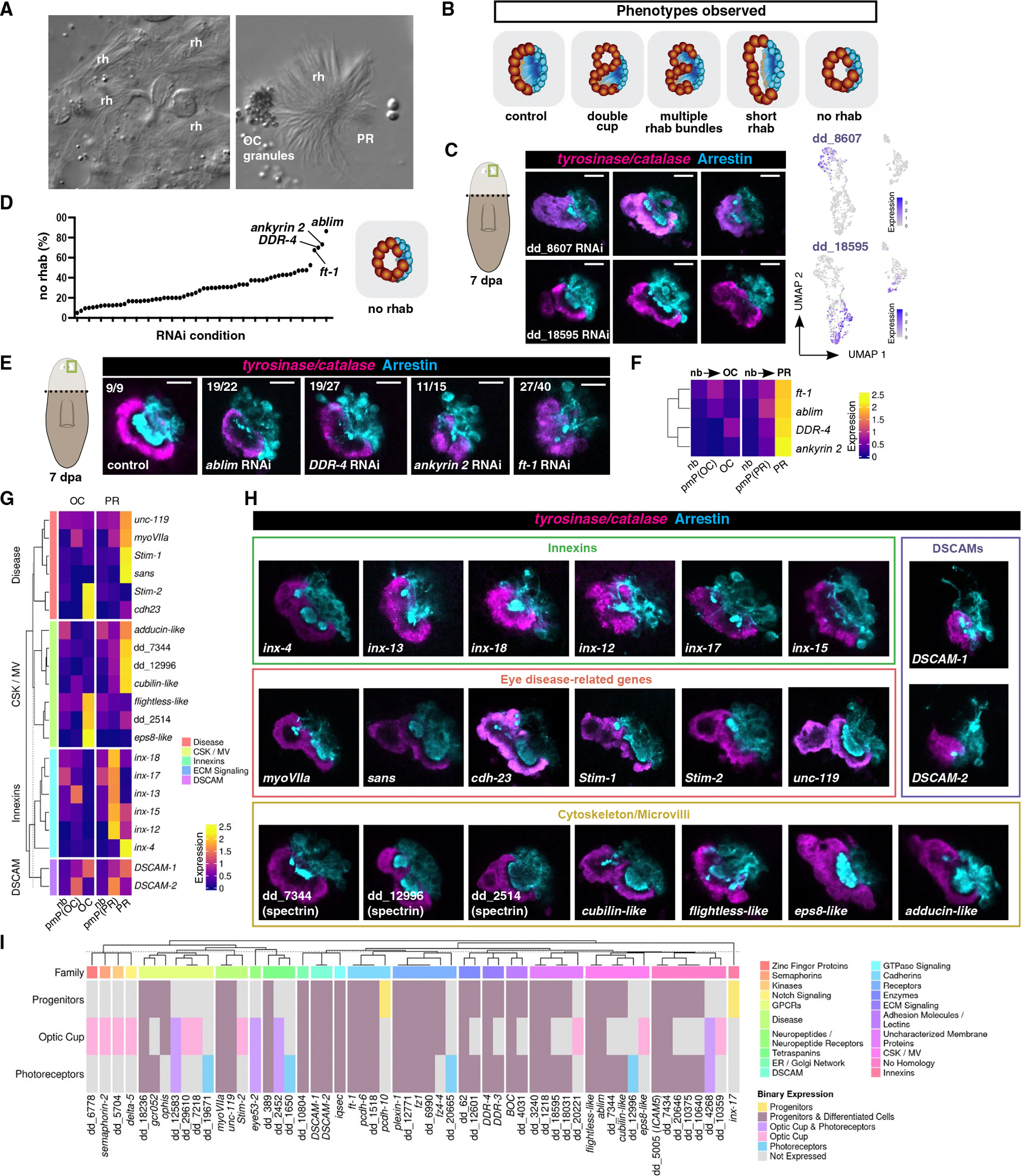
Genes required for rhabdomere formation and OC morphogenesis (A) DIC images showing rhabdomeres (rh) of a wild-type animal. (B) Cartoon shows the summary of morphological defects found throughout the RNAi screen. (C) FISH and immunostaining show examples of morphological eye defects in RNAi animals (left). UMAPs show gene expression (right). (D) Frequency of rhabdomere loss in regenerating RNAi animals at 7 dpa. (E) FISH and immunostaining show the lack of rhabdomere regeneration in RNAi animals. (F) Heatmap shows expression in the PR trajectory of genes required for rhabdomere formation. (G) Heatmap shows expression in PR and OC trajectories of gene families shown in (H) that caused eye morphological defects after RNAi. (H) FISH and immunostaining showing eye regeneration defects following RNAi. (I) Graph shows gene expression in progenitors, PRs, or OC cells of the genes that when inhibited caused eye morphological defects and affected phototaxis behavior. Cartoons of regenerating animals show region of images displayed. Scale bars, 20 μm in (C) and (E).

After eye progenitor differentiation, rhabdomere formation and extension initiate and coincide with OC cell aggregation, morphogenesis, and growth. Each PR rhabdomere bundle is an array of thousands of tightly packed apical membrane microvilli, where phototransduction occurs. These elaborate cellular structures are protrusions with actin bundles that increase the surface area of the cell, similar to other microvilli, such as the brush border of the intestinal epithelium, the proximal renal tubule, or the stereocilia in the hair cells of the inner ear.^46,47^ Planarian eyes present an attractive system to dissect the molecular activities that result in the formation of this understudied cellular elaboration because of their prominent morphology and accessibility. Planarian PR rhabdomeres are packed within the OC; following gentle dissociation, single PRs with microvilli can be observed using differential interference contrast (DIC) microscopy (Figures 4A and S4A). During regeneration, at around 4 dpa, OC cells and PRs interacted, and a small bundle of short rhabdomeres was apparent (Figure S4B). Analyses of FISH images during regeneration suggest that this interaction between both eye cell types might be the first step in the formation and extension of rhabdomeres.

Throughout the RNAi screen, we observed different types of morphological eye defects that we categorized as (1) double cup, (2) multiple rhabdomere bundles associated with an elongated or double OC, (3) short rhabdomere bundles, and (4) no rhabdomeres (Figure 4B and Table S3). Interestingly, many of these defects were observed following the inhibition of a single gene, suggesting that several of these defects can emerge as consequences of varying strength in the failure of particular biological phenomena associated with eye formation (Figure 4C). In principle, defects in Arrestin trafficking along the rhabdomeres rather than rhabdomere formation issues could be possible in some phenotypes. Moreover, these defects were observed following inhibition of genes either expressed exclusively in OC cells or in PRs (Figure 4C), pointing to the importance of interaction between both cell types in the acquisition of eye morphology.

Among the phenotypes where eyes regenerated with abnormal morphology (a phenotype was called when ≥50% of RNAi animals presented a defect), we assessed the frequency of animals that regenerated very short or no rhabdomeres at all. Four genes stood out, with the RNAi phenotype affecting rhabdomere formation with the highest penetrance among screened genes (Figures 4D, 4E, and S4C): *ablim*, *ankyrin 2*, a *discoidin domain receptor-4* (*DDR-4*), and *ft-1*. These RNAi animals also displayed aberrant phototaxis (Figure S4D). Following inhibition of each of these genes, regenerated OCs were mostly hollow, lacking or containing only very short rhabdomere projections (Figures 4E and S4C). PR numbers following RNAi were not overtly changed and axon projections were mostly normal (except for some *ankyrin 2* and *ablim* RNAi animals that lacked the optic chiasm, also pointing to roles for these genes in PR axon extension, Figure S4E).

UMAP plots, trajectory analysis, and FISH showed that expression of all four genes was enriched in PRs and their progenitors (Figures 4F, S1L, and S4F). Ablim contains two LIM domains and a C-terminal VHP (villin headpiece) domain, which is involved in F-actin bundling in cytoskeletal proteins (Figure S4F). Ablim family members bind actin filaments and are expressed in retina and brain tissues in several species.^48,49^ Ankyrin 2 contains multiple ankyrin repeats (Figure S4F), common in scaffolding proteins and intermicrovillar linkers.^47^ *ft-1* encodes a homolog of Fat, an atypical cadherin, and this gene was previously shown to be required for the establishment of ciliary rootlet polarity in planarian epidermal cells.^50^ Finally, *DDR-4* encodes a homolog of discoidin domain receptors, which are collagen-activated tyrosine kinases (Figure S4F).

Of all the genes inhibited in this RNAi screen, 145 RNAi conditions resulted in eye morphogenesis defects, indicating a complex array of molecular activities is important for eye-structure formation. Within this collection, there were multiple genes encoding members of particular protein families (e.g., innexin, DSCAM, tetraspanin, cadherin, and GPCR), proteins with related functional properties (e.g., proteins that interact with the extracellular matrix (ECM) or cytoskeleton/microvilli components), or proteins involved in signaling pathways (Notch). Furthermore, multiple of these genes have homologs associated with eye diseases in humans, such as Usher syndrome (i.e., *myoVIIa*, *sans*, and *cadherin 23*), corneal dystrophies and Stormorken syndrome (i.e., *Stim* genes), and cone-rod dystrophies (i.e., *unc-119*) (Figures 4G, 4H, S4G, and S4H and Table S3). There were numerous other genes for which inhibition caused morphological eye defects that encode proteins with no homology (19/145) and proteins with homologs in humans but that remain uncharacterized (13/145), or where no prior mechanistic connection to eye biology exists. We functionally examined the negative phototaxis behavior of RNAi animals harboring these defects, and many were required for normal eye function (Figures 4I and S4I and Table S3). This work identifies a large collection of proteins involved in this phase of eye regeneration, with some required for the formation of rhabdomere projections and others for OCs to develop stereotyped architecture and form, providing a resource for investigating the gene functions in eye biology, including orthologs of genes associated with human eye disease.

### Regeneration of form: DDR-1 and a calcium transporter are required for eye transparency and formation of the dorsal projection field

**Figure 5.**
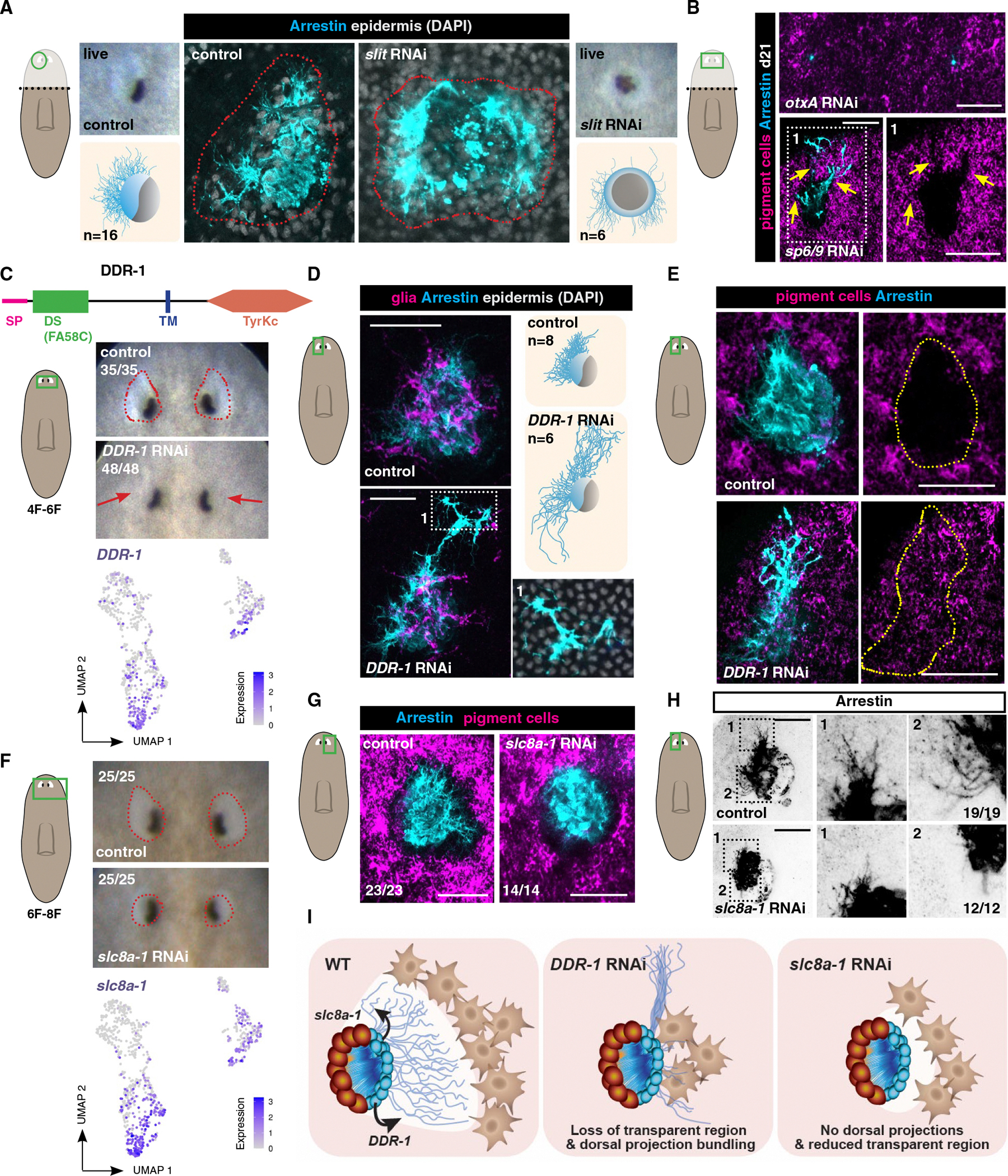
*DDR-1* and *slc8a-1* are required for eye TR formation (A) Immunostainings show stereotypical dorsal projection arborization in control (left) or *slit* RNAi (right) animals. Cartoons show dorsal projection tracings in each condition. (B) FISH and immunostaining show a lack of transparent region (TR, no exclusion of pigment cells) in an *otxA* RNAi animal (top) and correlation of TR with dorsal projection bundles (yellow arrows) in a *sp6/9* RNAi animal (bottom). (C) DDR-1 protein structure (top), live images showing lack of TR in a *DDR-1* RNAi animal (middle), and UMAP plot showing *DDR-1* expression in eye cells (bottom). (D) FISH and immunostaining show dorsal projection bundling (cyan) and normal glia distribution in a *DDR-1* RNAi animal. Inset shows dorsal projections toward the epidermis (DAPI, nuclei). Cartoons show dorsal projection tracings (top right). (E) FISH and immunostaining show pigment cells are juxtaposed to PR cell bodies (cyan) in a *DDR-1* RNAi animal. (F) Live images (top) show reduced TR in a *slc8a-1* RNAi animal. UMAP plot (bottom) shows *slc8a-1* expression in the eye. (G) FISH and immunostaining show reduced TR in an *slc8a-1* RNAi animal. (H) Loss of PR dorsal projections in a *slc8a-1* RNAi animal. (I) Drawings summarize the phenotypes observed. Red dotted lines and arrows show TR. Yellow dotted lines outline eye position. Cartoons of regenerating (A and B) or uninjured (C–H) animals show the region of images displayed. Scale bars, 50 μm.

After eye progenitor specification, nucleation, and differentiation as well as the regeneration of OC form and PR rhabdomeres, the next step in eye regeneration involves the formation of dorsal PR projections and interactions with the surrounding environment to produce an eye transparent zone associated with light passage. Planarian PRs have dorsal projections with unclear function, a distinction from mammalian cones and rods. These projections and their arborization became apparent relatively late in the eye regeneration process (10–15 dpa, Figure S1A). The dorsal projection field had a highly stereotyped shape, with projections extending anteriorly, laterally, and dorsally (Figure 5A). These dorsal projections avoid the posterior eye region as well as the midline, potentially being repelled by Slit (see below). The dorsal projections coincide with the transparent, or white, part of the planarian eye (Figure 5A). This white/TR gives many planarian species their stereotypical cartoon-like eye appearance. The correlation between dorsal projection presence and eye TR was apparent following *slit* RNAi, where the regenerating cyclopic eye formed at the midline, as previously shown,^51^ had dorsal projections in all directions and a TR surrounding the eye in a circular shape (Figure 5A).

The formation of the TR requires PRs. Animals that could not specify PRs (i.e., *otxA* or *klf* RNAi animals) did not regenerate the TR (Figure 5B) and displayed a very reduced TR in uninjured animals, associated with failure to maintain the PR population during tissue turnover (Figure S5A). In *sp6/9* RNAi animals that cannot specify OC cells and have disorganized PRs, dorsal projections bundle and project toward the dorsal and anterior (ectopically far anterior, Figure S5B). Under this condition, the TR, shown by FISH as the absence of body pigment cells, matched the aberrant and irregular projection pattern of dorsal bundles (Figure 5B). These observations suggest a functional connection (further explored below) between dorsal projections and the formation of a TR of particular shape and scale for light transmission to the planarian eye.

From all genes inhibited in the screen, inhibition of only one gene—*DDR-1* (previously named *ddryk-1*^4^)—caused complete TR loss during homeostatic eye maintenance without affecting PR specification (Figures 5C–5E). The rarity of this phenotype points to specific molecular processes in the generation of this transparent, patterned region. *DDR-1* encodes a discoidin domain tyrosine kinase, and it was mostly expressed in PRs and their progenitors (Figures 5C and S1L). Strikingly, *DDR-1* RNAi animals lacked the stereotypical shape of the PR dorsal projection field. Instead, dorsal projections were bundled and extended anteriorly and dorsally toward the epidermal layer (Figure 5D). This phenotype bears similarity to that described above for regenerating *sp6/9* RNAi animals lacking OCs (Figure S5B); however, in this case, OCs were present.

Body pigment cells are normally excluded from the planarian eye TR.^15^ However, in *DDR-1* RNAi animals, pigment cells were seen juxtaposed next to PR cell bodies (Figure 5E), indicating that a component in the TR is able to repel or block the presence of pigment cells and their extensions. Depigmented animals, which have a reduced number of body pigment cells, achieved with photoablation,^15^ did not show any defect in dorsal projection morphology, suggesting that pigment cells themselves do not affect PR dorsal projection behavior (Figure S5C). Unlike sp*6–9* RNAi animals, where pigment cells still avoided the ectopic anterior projection bundles, the disorganized and ectopic dorsal-anterior projections of *DDR-1* RNAi animals were interspersed with pigment cells (Figure 5E). This indicates that *DDR-1* has a specific role in dorsal projection patterning and in the generation of the TR by dorsal projections.

Homeostatic turnover of planarian eyes is constant, and progenitor cells continually incorporate into the eye, replacing the pre-existing cells as part of normal adult life.^4,5^ Early *ovo*+ eye progenitors are mostly found in the prepharyngeal region of the animal, far from the eyes.^4^ It has previously been shown that eyes transplanted into this region can be maintained indefinitely through new progenitor incorporation.^6^ We performed transplantation experiments to further assess the properties of the eye TR. We transplanted wild-type eyes (containing the TR) into prepharyngeal animal regions of wild-type recipients and subsequently performed RNAi of *DDR-1*. *DDR-1* RNAi caused TR loss in the transplanted ectopic eye (Figure S5D). This indicates that it is the eye itself and the role of *DDR-1* in the eye that sets the location of the TR, as opposed to some independent patterning process with cues that coincide with the normal location of the eye. This is also consistent with a variety of RNAi phenotypes where ectopic eyes also present TRs.^6,8,52^ In addition, transplanted *klf* RNAi eyes (which originally do not have a TR because of a lack of PRs) into wild-type recipients developed the TR over time, coincident with wild-type PR progenitors from the recipients incorporating into the transplanted eyes (Figure S5D).

DDR proteins are single-pass transmembrane receptor tyrosine kinases (RTKs), but unlike many RTKs that signal following growth factor ligand binding, they interact with the ECM by binding to collagen through a discoidin (DS) domain. We investigated the expression of matrisome genes^53^ in the planarian eye (Figure S5E), inhibited some of these ECM genes and most planarian collagen genes by RNAi (20 genes total, Figure S5F and Table S3), and assessed the eye TR but did not observe TR changes. Therefore, it remains unclear whether planarian *DDR-1* has a collagen-independent activation or whether there is redundancy among multiple collagen genes for this role. Vertebrate DDRs can regulate the expression of cadherins, integrins, and other ECM-interacting proteins to modulate cell-matrix adhesion.^54–56^ They can also control ECM remodeling through the regulation of matrix metalloproteinase (MMP) expression and activity.^57,58^ To examine whether *DDR-1* regulates gene expression in planarian eyes, we performed bulk mRNA sequencing of head fragments of control and *DDR-1* RNAi animals. We inhibited several candidate genes (12 genes, Figure S5G and Table S3) encoding proteases, peptidase inhibitors, and genes involved in heparan sulfate glycan synthesis or lipid binding, that were downregulated in *DDR-1* RNAi animals and expressed in our 10× eye dataset. We were unable to detect changes in the eye TR following RNAi of the candidate genes. In addition, we isolated eyes from control and *DDR-1* RNAi animals and performed a proteomics analysis. We found a few candidate genes encoding proteins that were downregulated in *DDR-1* RNAi and expressed in the planarian eye. The RNAi screen of several of these genes (11 genes, Figure S5H and Table S3) did not uncover genes required for eye TR formation and maintenance.

Regenerating eyes in *DDR-1* RNAi animals showed a similar phenotype to that observed in uninjured RNAi animals, with dorsal bundles projecting anteriorly and dorsally (Figure S5I). Eye-associated glia appeared normal under this RNAi condition in both regeneration and homeostasis, indicating that glia are not driving the presence of the TR (Figures 5D and S5I). The eye-associated TR did not regenerate in *DDR-1* RNAi animals, and pigment cells were present next to the PRs (Figure S5J).

Inhibition of another gene that is expressed in PRs and their progenitors, *slc8a-1*, which encodes a Na^+^/Ca^++^ transporter,^59^ caused a partial reduction of the eye TR (Figure 5F). FISH analyses showed that pigment cells were closer to the PR cell bodies in *slc8a-1* RNAi animals than in controls (Figure 5G). Interestingly, immunostaining with an anti-Arrestin antibody showed a marked reduction in dorsal projection arborization in these RNAi animals (Figure 5H). Axonal projections from PRs were not affected in *slc8a-1* RNAi animals, suggesting that the effect was specific to dorsal projection extension/branching (Figure S5K). These RNAi animals also showed phototaxis behavioral defects (Figure S5L). Taken together, our data show that the formation of the eye TR is dependent on the presence of PRs and is strongly correlated with dorsal projection morphology. TR development involves activity intrinsic to the eye (i.e., not dependent on the position/location of the eye) and is regulated by the *DDR-1* gene. Our data also suggest that arborization of dorsal projections likely facilitates the expansion of the TR (further away from the PR soma), creating an area for light transmission (Figure 5I).

### Progenitor incorporation: A surface molecule signature mediates eye progenitor trapping by eyes

During the growth of the regenerating eye and during normal tissue turnover thereafter, migratory eye progenitors are continuously incorporated into the eye. As noted above, eye progenitors are specified coarsely, sometimes 10s–100s of microns from the eyes (in the prepharyngeal region). These progenitors migrate to the right location in the head (the target zone) for nucleation and differentiation following extrinsic cues. Notably, ectopic or transplanted eyes in the *ovo*+ progenitor specification zone (away from the normal progenitor target zone) also incorporate *ovo*+ progenitors that differentiate to successfully maintain the ectopic eyes through a self-organizing process. This suggests that in addition to the extrinsic information at the target zone, eye-intrinsic mechanisms also act to capture progenitors and promote their differentiation.^6,8^

**Figure 6.**
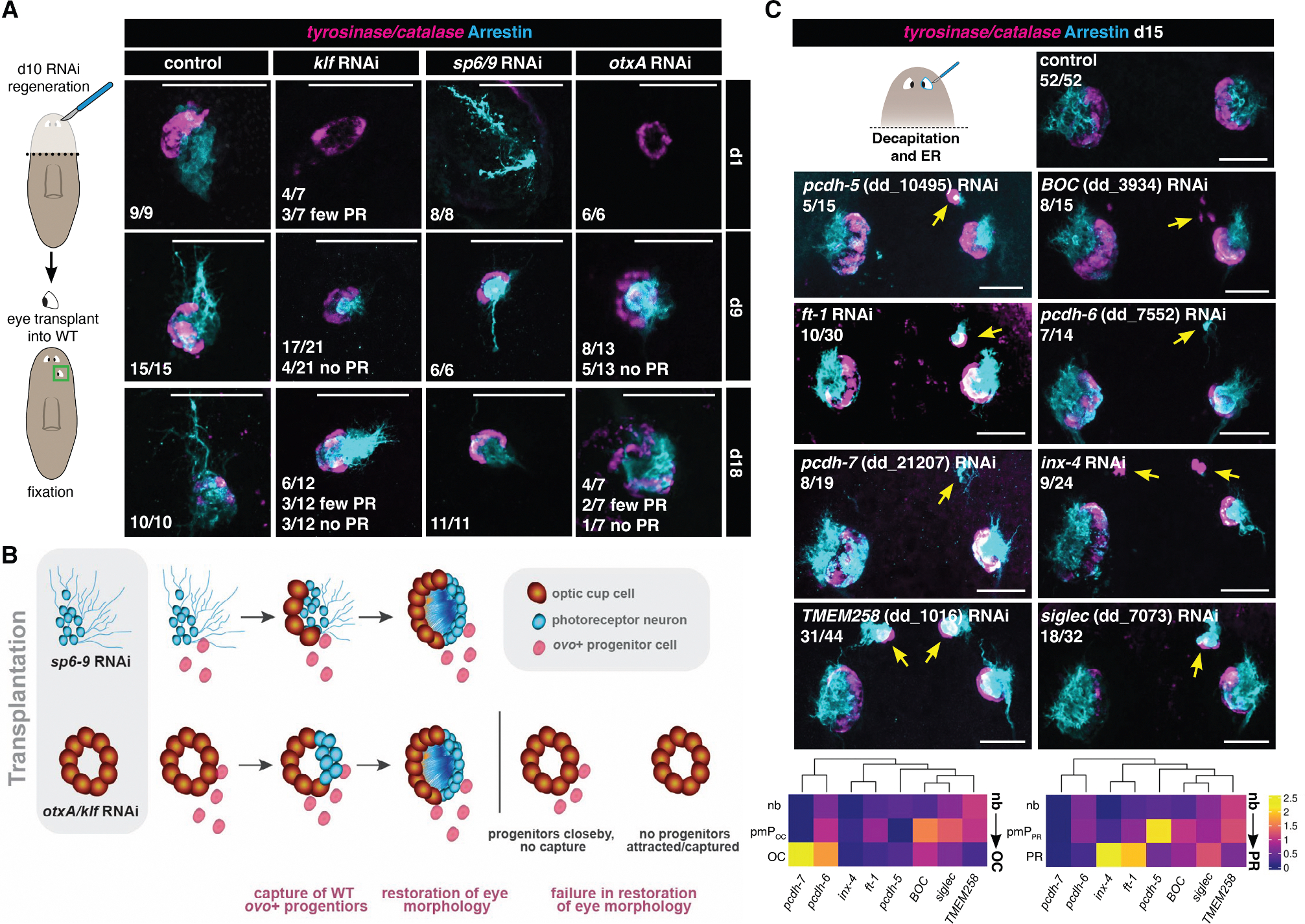
Genes involved in eye progenitor trapping (A) FISH and immunostainings show eye morphology and cell-type composition at different time points following eye transplantation. (B) Drawings summarize the transplantation findings. (C) FISH and immunostainings show defects in progenitor trapping following eye resection in a morphallaxing head fragment. Yellow arrows point to ectopic eyes. Heatmaps (below) show expression of the genes involved in trapping throughout the PR and OC trajectory. Cartoons show surgical procedures. Scale bars, 100 μm in (A) and 50 μm in (C).

To better understand this self-organizing process involving progenitor trapping by eyes, we first addressed whether eyes comprised of only one cell type (PRs or OC cells) were able to attract and capture eye progenitors when transplanted into the prepharyngeal region of a wild-type animal. We transplanted eyes from regenerating *klf* and *otxA* RNAi animals (no PRs) or *sp6/9* RNAi animals (no OC cells) into wild-type recipients. OC- or PR-only eyes were both able to interact with and incorporate eye progenitors from the host, eventually forming eyes of normal appearance over time (Figure 6A). However, a fraction of transplanted OC-only eyes was unable to capture progenitors and remained with only OC cells even at 18 days post-transplantation (Figure 6A). It is possible that the closed, spherical OC morphology of these eyes interfered with eye progenitor capture or that PRs are more efficient than OC cells at capturing progenitors (Figure 6B).

We reasoned that an approach to identify molecules with roles in capturing eye progenitors could involve decapitating RNAi animals and resecting one eye (unilaterally) at the time of decapitation in the head fragments (0 dpa). After 2 to 3 dpa, positional information will shift in these head fragments to direct regeneration in the small-sized fragments. During this rescaling process, the position of the target zone for eye nucleation changes, shifting anteriorly along with the shifting positional information.^6,8^ However, because the eye was resected at 0 dpa and positional information takes 2 to 3 days to shift, a new small eye will nucleate near the original eye location and grow in place by trapping progenitors despite the anterior movement of the eye target zone. This challenges the self-organizing capacity of the eye by requiring a small, nucleated eye to trap progenitors that would otherwise prefer to move more anteriorly. Inhibition of genes that are important for the capture of progenitors might therefore cause a second eye to form anteriorly in this injury context.

Whereas control RNAi animals never formed a second anterior eye (0/52), RNAi animals following inhibition of select genes did (Figure 6C). Similar to the results observed following transplantation, *klf* and *otxA* RNAi head fragments showed mature OC cells anterior to the regenerating eye following eye resection, further suggesting that OC-only eyes are less efficient at capturing progenitors (Figure S6A). In addition, inhibition of four different genes encoding cadherin family members (*ft-1* and the protocadherin genes *pcdh-5*, *pcdh-6*, and *pcdh-7*), resulted in the formation of anterior second eyes (Figure 6C). This points to important roles for cadherin-mediated cell-cell interactions in the formation of eye architecture from dispersed progenitors. We also found that inhibition of three other genes encoding proteins similar to the human orthologs—brother of contactin, BOC (dd_3934); TMEM258 (dd_1016); and Siglec (sialic acid binding Ig-like lectin, dd_7073)—resulted in the formation of a second anterior eye or anterior differentiation of mature eye cells in the rescaling head fragments (Figure 6C). Sequence similarities to the human orthologs were low for these proteins, but 3D structure predictions using AlphaFold were very similar (Figure S6B). Moreover, inhibition of the innexin *inx-4* gene also resulted in ectopic anterior eyes in head fragments. Interestingly, while some of these genes were expressed exclusively in differentiated PRs (*inx-4* and *ft-1*) or OC cells (*pcdh7*), most of them were expressed in eye pmPs (Figures 6C and S6C). Finally, inhibition of two genes encoding frizzled proteins, *fz1* and *fz4–4*, and the netrin receptor gene, *unc5a*, also resulted in the regeneration of a second anterior eye in this context (Figure S6D). None of the animals in these RNAi groups regenerated an eye with a clear anterior shift in location following eye resection in uninjured animals (Figure S6E), pointing toward roles for these genes in the biology of eye progenitors and mature eye cells for self-organization of the eye. An additional select group of genes for which RNAi also caused the formation of a second anterior eye, but with distinguishing properties, will be discussed in the next section (see below).

In many organisms, progenitor incorporation into tissues is an essential step in development and homeostasis.^60^ Here, we found a group of cadherin-family adhesion molecules, as well as genes encoding a contactin-like protein (*BOC*), a lectin (*siglec*), and a *TMEM258*-like gene that are required for efficient trapping of eye progenitors by the eye. The *TMEM258* gene is predicted to be involved in glycosylation and might modify different membrane proteins required for cell adhesion. The molecules uncovered here present an opportunity for the dissection of how surface-molecule signatures promote a self-organizing process involving interactions between progenitors and mature differentiated cells during organ regeneration.

### Progenitor incorporation: Several DDRs are required for localized arborization of dorsal projections and progenitor trapping

**Figure 7.**
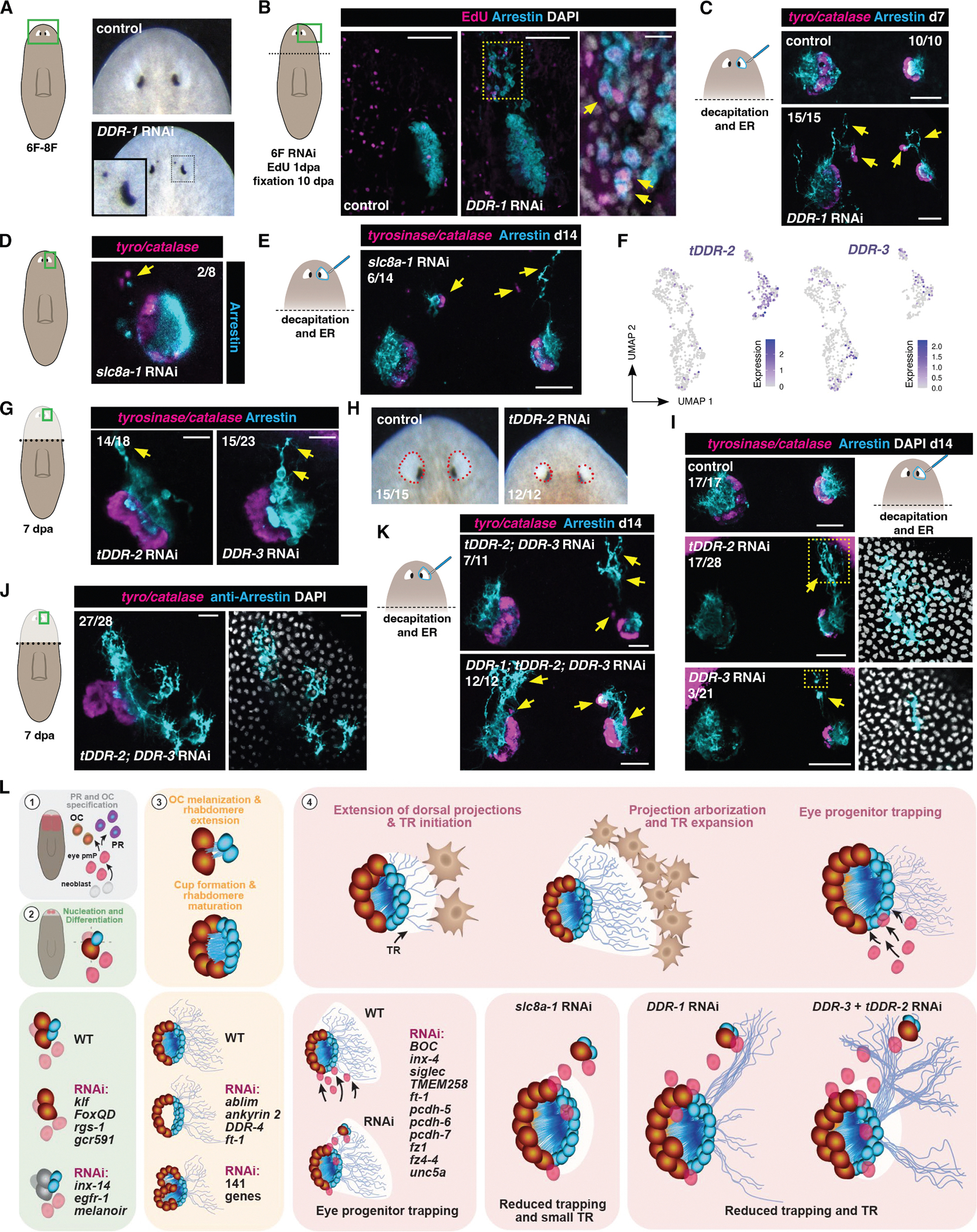
*DDRs* and *slc8a-1* are required for dorsal projection arborization and progenitor trapping (A) Ectopic eyes in an uninjured *DDR-1* RNAi animal. (B) FISH and immunostaining show newly made EdU+ cells (yellow arrows) in the ectopic eye (dotted inset) of a *DDR-1* RNAi animal. (C) Ectopic anterior eyes (yellow arrows) in a *DDR-1* RNAi head fragment. (D) Differentiated ectopic cells (yellow arrow) in an uninjured *slc8a-1* RNAi animal. (E) Ectopic anterior eyes (yellow arrows) in a *slc8a-1* RNAi head fragment. (F) *tDDR-2* and *DDR-3* gene expression in the eye. (G) PR disorganization (yellow arrows) in regenerating *tDDR-2* and *DDR-3* RNAi animals. (H) Live images show reduced TR in an uninjured *tDDR-2* RNAi animal. (I) Ectopic anterior eyes (yellow arrows) in morphallaxing head fragments of *DDR* gene family RNAi animals. Insets show dorsal projections extending toward the epidermis (DAPI). (J and K) Stronger defects in progenitor trapping and dorsal projection bundling show redundancy of *DDR* gene family members in a regenerating animal (J) and in a morphallaxing head fragment (K) following combined RNAi of *DDR* family genes. (L) Model summarizing results through eye regenerating stages (organized into four temporal phases). Cartoons of uninjured (A, D), morphallaxing (B, C, E, I, and K), or regenerating (G and J) animals show the region of images displayed. Scale bars: 50 μm and 10 μm in (B inset).

The requirement for *DDR-1* in forming the eye TR was described above. Inhibition of *DDR-1* also caused the formation of ectopic eyes in uninjured animals after six to eight dsRNA feedings (Figure 7A). 5-ethynyl-2’-deoxyuridine (EdU) incorporation assays showed that ectopic anterior eyes in these animals were mostly formed by newly differentiated eye cells (EdU+ cells) (Figure 7B). Eye regeneration following unilateral eye resection in uninjured *DDR-1* RNAi animals occurred at the target zone (the same position as the original eye, Figure S7A), indicating that ectopic eyes were not the result of a target zone shift after RNAi. The *DDR-1* RNAi phenotype, therefore, differs from previously studied position control gene (PCG) RNAi conditions that can cause homeostatic duplications of eyes, where the target zone shifts and the eyes form at new target zone positions (e.g., *ndk* and *notum* RNAi^6,8,52^). In addition, *DDR-1* inhibition resulted in the formation of a second pair of anterior eyes following unilateral eye resection in morphallaxing head fragments (Figures 7C and S7B), indicating a role for *DDR-1* in eye progenitor trapping.

Similar to the phenotype described above, inhibition of *slc8a-1* in uninjured animals also resulted in eye progenitors escaping the influence of the eye and differentiating anteriorly (Figure 7D). Unilateral eye resection in morphallaxing *slc8a-1* RNAi head fragments also resulted in the formation of anterior ectopic eyes (Figure 7E). However, similar to *DDR-1* RNAi animals, a new eye regenerated at the same location as the original eye following a unilateral eye resection in *slc8a-1* uninjured RNAi animals (Figure S7C), indicating that the ectopic second eye observed in head fragments was a consequence of an eye-intrinsic failure in eye progenitor trapping and not a consequence of target zone shifts. Altogether, these data suggest that the eye region associated with the TR contributes to eye progenitor-trapping by the eye.

DDR proteins are found broadly in the animal kingdom, from pre-bilaterians such as cnidarians and sponges to humans.^58^ These ancient metazoan proteins might have central roles in the production of tissue architecture and boundaries.^54,61–63^ In this study, we described a role for *DDR-4* in rhabdomere formation (Figure 4D) and for several members of the DDR family in eye morphogenesis (Figure S4G) and an additional role for *DDR-1* in dorsal projection morphology, TR formation, and progenitor trapping (Figures 5 and 7). The roles of the broadly conserved DDR family in animal regeneration are previously unknown. Our results suggest that this gene family will prove to have widespread roles in the maintenance and regeneration of adult tissue architecture. Given these considerations and the fundamental requirements for *DDR* family genes in planarian eye biology, we sought to characterize the entire *DDR* gene family in the planarian genome. We found six DDRs (*DDR-1–6*) and noted that there were also multiple (three) genes encoding truncated DDR proteins lacking a kinase domain, similar to vertebrate genomes; we named these truncated genes *tDDR-1– tDDR-3* (Figure S7D). Four of the six *DDRs* (*DDR-1–4*), as well as all *tDDRs*, were expressed in the planarian eye (Figures 7F and S7D). Inhibition of either *DDR-3* or *tDDR-2* caused PR disorganization with anterior dispersion of PRs in regeneration (Figure 7G). In addition, *tDDR-2* RNAi animals displayed a reduced eye TR (Figure 7H). Unilateral eye resection in *tDDR-2* or *DDR-3* RNAi head fragments resulted in the differentiation of ectopic PRs and bundles projecting anteriorly and dorsally to the epidermal layer, similar to the phenotype observed in *DDR-1* RNAi animals (Figure 7I). Similar to the results observed in *DDR-1* RNAi animals, eye regeneration following eye resection in uninjured animals occurred at the original location, indicating no changes in positional information related to the eye target zone occurred in these RNAi animals (Figure S7E). Inhibition of *DDR-2* caused some eye morphological defects (Figure S7F) but had no effect on dorsal projection morphogenesis or eye progenitor trapping (Figure S7G). Similarly, no defects were observed following inhibition of *tDDR-3* (Figures S7F and S7G). Altogether, these findings point to the importance of multiple DDRs in eye progenitor trapping, arborization of dorsal projections, and formation of the eye TR.

Given the roles of multiple *DDR* genes in progenitor trapping and TR formation, we wondered whether there might be partially redundant roles for some *DDR* family members. Double inhibition of *tDDR-2* and *DDR-3* caused a much stronger and dramatic phenotype with 100% penetrance. All *tDDR-2; DDR-3* RNAi animals showed differentiated PRs outside of the target zone as early as 7 dpa, with bundling of dorsal projections toward the epidermal layer Figure 7J). Similarly, anterior and lateral differentiated PRs formed in morphallaxing head fragments after unilateral eye resection in double *tDDR-2; DDR-3* RNAi animals (Figure 7K). These defects were even stronger following inhibition of *DDR-1*, *tDDR-*2, and *DDR-3* (Figure 7K). Taken together, our findings suggest that dorsal projections are required to expand the eye TR and help with progenitor trapping into the intact eye for its maintenance. Moreover, we showed that several members of the DDR family act redundantly to facilitate TR formation and eye progenitor trapping into the eye.

## DISCUSSION

In this study, we explored the sequential steps that lead to the regeneration of the planarian eye. We described a hierarchical program for eye regeneration in planarians with an array of genes required at each stage of the process (Figure 7L). A number of identified molecular processes are also required for the homeostatic maintenance of the eye. We isolated planarian eyes; performed scRNA-seq of OCs, PRs, and eye progenitors; inhibited the expression of a total of 480 genes through an RNAi regeneration screen; and performed a systematic functional behavioral assay to determine the ability of regenerating RNAi animals to respond to light. A total of 164 genes had roles in eye regeneration and maintenance (Table S3). This work developed an extensive resource with information on the expression of all genes in eye progenitor differentiation trajectories and phenotypes affecting different stages of regeneration, including genes encoding signaling molecules, TFs, regulators of ECM organization, orthologs of eye-disease genes, and conserved factors. The database is accessible at: https://digieye.wi.mit.edu.

We found several genes and functions for previously described genes involved in the initial specification and differentiation of eye progenitors into PRs (*FoxQD*, *gcr591*, *klf*, and *rgs-1*) and for three genes (*inx-14*, *melanoir*, and *egfr-1*) involved in later maturation processes (i.e., melanin production for OC cells). A large number of genes (145), including those encoding orthologs associated with eye disease, adhesion molecules, ECM or cytoskeleton, and GPCRs, as well as genes with orthologs with no prior known function in eye biology, were identified to be involved in the morphogenesis of the planarian eye. Of these 145 genes, 61 were essential for normal negative phototaxis. We found a small cohort of genes required for rhabdomere formation (*ablim*, *ankyrin 2*, *ft-1*, and *DDR-4*) and propose a potential mechanism for these microvilli-type projections to extend, in a process involving an initial interaction between PRs and OC cells. We identified a Ca^++^ transporter-encoding gene *slc8a-1*, as essential for PR dorsal projection formation and arborization. We also described molecules (DDR family members) required for the formation and expansion of PR dorsal projections and the eye TR. We found a role for PR dorsal projections in progenitor trapping by the eye, highlighting a potential evolutionary advantage for this attribute of planarian PRs. Moreover, combining RNAi experiments with eye transplantation and surgical eye removal techniques, we found cellular and molecular requirements (including roles for 15 genes, such as those encoding DDR proteins and protocadherins) for eye progenitor trapping, an essential process required for the regeneration and maintenance of this organ. How surface molecule signatures result in self-organizing processes by which progenitors and mature cells organize into spatial patterns is a fundamental but poorly understood problem. In one example process also involving cadherins, different zebrafish cadherin classes similarly facilitate spatial patterning of neural tube cell types from an initial noisy pattern of progenitors.^60^ Our data also suggest that PRs are more efficient at trapping eye progenitors than OC cells.

Our results shed light on the prominent role of an underexplored family of genes conserved across the metazoa, the *DDRs*, at different stages of eye regeneration and morphogenesis. Whereas *DDR-4* was central for rhabdomere formation, *DDR-1*, together with the truncated receptor *tDDR-2*, was crucial for the maintenance and regeneration of the eye TR. In addition, we found that *DDR-3* acts redundantly with *tDDR-2* and *DDR-1*, with these molecules being essential for eye progenitor trapping and preventing ectopic eye cell differentiation. DDRs have been shown to have roles in modulating matrix stiffness, a process that involves crosslinking of ECM proteins, regulation of MMP and ADAM metalloproteinases, and excessive deposition of ECM components, which subsequently modifies cell behavior.^64^ Matrix stiffness has also been linked to tumor aggression and cancer progression.^65^ Moreover, in *C*. *elegans*, disruption of a DDR homolog affects tissue basement membranes and the connections between adjacent tissues.^61^ The maintenance of these tissue boundaries and a balance in ECM rigidity are essential for cellular homeostasis and tissue architecture. Our data support the potential role of planarian DDRs in regulating and modifying the ECM to generate a TR that might facilitate light sensing as well as facilitating the trapping of regenerative progenitors.

Some of the genes described in this study belong to specific gene families that have not been previously linked to eye biology or that are uncharacterized in other systems but might play key roles in vertebrate eye formation and human eye diseases. Our approach identified genetic features required in a hierarchical program for planarian eye regeneration, defining cellular and molecular steps for the regeneration of an animal organ.

### Limitations of the study

Inhibition of gene expression by RNAi might not have been sufficient for detecting phenotypes for some genes studied. Additionally, not all genes expressed in planarian eyes were studied. Therefore, there might exist genes in addition to the ones described in this study that are required for the stages of planarian eye regeneration investigated here. It is possible that, because of the low numbers of pMPs (OC) captured in this study, the transcriptome of those cells does not have optimal accuracy. Future studies could investigate those particular pmPs more fully. Immunostaining with the anti-Arrestin antibody does not always allow distinction between rhabdomeres and dorsal PR projections, presenting some limits on projection characterization in RNAi cases. Behavioral assays analyzed in this study only tested negative phototaxis and, therefore, future work will be important to assess the role of certain genes in other behavioral assays.

## RESOURCE AVAILABILITY

### Lead contact

Requests for further information and resources should be directed to and will be fulfilled by the lead contact Peter W. Reddien (reddien@wi.mit.edu).

### Materials availability

This study did not generate new, unique reagents.

### Data and code availability

- The eye scRNA-seq data generated in this study have been deposited at the Sequence Read Archive (SRA) under the accession number SRA: PRJNA1269575. Additionally, this paper analyzes existing, publicly available data, accessible at SRA: PRJNA1067154.
- This paper does not report original code.
- Any additional information required to reanalyze the data reported in this paper is available from the lead contact upon request.

## STAR★METHODS

### EXPERIMENTAL MODEL AND STUDY PARTICIPANT DETAILS

Asexual *Schmidtea mediterranea* strain animals (CIW4) were cultured in 1x Montjuic planarian water (1.6 mmol/L NaCl, 1.0 mmol/L CaCl_2_, 1.0 mmol/L MgSO_4_, 0.1 mmol/L MgCl_2_, 0.1 mmol/L KCl and 1.2 mmol/L NaHCO_3_ prepared in Milli-Q water) at 20°C. Animals were starved 1–2 weeks prior to experiments.

### METHOD DETAILS

#### 10x single-cell mRNA sequencing

Head blastemas of regenerating day 3 and day 5 trunk pieces where surgically isolated, incubated in ice-cold CMF (400 mg/L NaH_2_PO_4_, 800 mg/L NaCl, 1200 mg/L KCl, 800 mg/L NaHCO_3_, 240 mg/L glucose, 15 mM HEPES, pH7.3) solution with 1% BSA (CMFB) and 1 mg/mL of collagenase for 10 min at RT with gently pipetting. Samples were then incubated with 1:50 dilution of Hoescht (10 mg/mL) for 45 min at RT. X1 and X2 cells were then isolated by flow cytometry as described.^79^ Head pieces containing eyes were gently amputated for dissociation using a surgical scalpel and incubated in trypsin at RT for 10 min. The pieces were gently pipetted to isolate full pure eyes. The eyes were picked with a P100 pipette and placed in ice-cold DPBS without calcium or magnesium (Gibco, Thermo-Fisher Scientific, Cat. No: 14040117). Isolated eyes were then briefly washed with DPBS without calcium or magnesium (Gibco, Thermo-Fisher Scientific, Cat. No: 14190250), placed in Papain Dissociation System solution (Worthington Biochemical Corporation, Cat: LK003150), prepared in Neurobasal Medium (Gibco, Thermo-Fisher Scientific, Cat. Num.: 21103049) supplemented with B-27 Plus Supplement (Gibco, Thermo-Fisher Scientific, Cat. Num: A3582801), and incubated in a 34°C water bath for 20 min. Following gentle pipetting, to dissociate the eyes into single cells, a second 6-minute-long incubation, and additional pipetting were performed. Papain activity was inhibited using an Ovomucoid Inhibition Solution (prepared in EBSS solution) following the manufacturer’s recommendations. The samples were then centrifuged at 500 g for 5 min, resuspended in CMFB and filtered using a 40μm filter. Samples were counted with Trypan blue to determine the optimal cell number needed for the 10X single-cell sequencing procedure. Each condition was run as a single sample in 10X library preparation. Cells were processed by the WIGTC core (Whitehead Institute for Biomedical Research, Genome Technology Core) using 10X Genomics Chromium Controller and Chromium single cell 3^′^ Library & Gel Bead Kit (PN 1000006) following standard manufacturer’s protocol. Samples were sequenced on an Illumina NovaSeq 6000 (150 × 150 paired-end reads) across all lines. Sequencing reads were mapped using a GTF file of Smed_v6 (https://planmine.mpinat.mpg.de/planmine/model/bulkdata/dd_Smed_v6.pcf.contigs.fasta.zip) genes in the context of the Smes_g4 (https://planmine.mpinat.mpg.de/planmine/model/bulkdata/dd_Smes_g4.fasta.zip) genome. This GTF file was generated by using BLAT to map all Smed_v6 transcripts to the Smes_g4 genome and each transcript was assigned to a single genome location based on the best alignment score. Transcripts were then collapsed using genome location before mapping using the 10X Genomics Cell Ranger 7.2.0 pipeline. Cells were assessed for nUMI, nGene, and percent mitochondrial transcript content, which was represented in violin plots. Percent mitochondrial content was based on mitochondrial genes previously reported^80^ which are represented in v_6 of the Dresden transcriptome (dd_Smed_v6_258_0_1, dd_Smed_v6_289_0_1, dd_Smed_v6_292_0_1, dd_Smed_v6_297_0_1, dd_Smed_v6_344_0_1, dd_Smed_v6_505_0_1, dd_Smed_v6_753_0_1, dd_Smed_v6_957_0_1) and on the highly abundant mitochondrial transcripts (mtRNA_1, mtRNA_2).^81^ Doublets were identified via scDblFinder (https://bioconductor.org/packages/release/bioc/html/scDblFinder.html) and removed after basic QC filtering; any cells with nFeature_RNA < 750, nFeature_RNA > 3000, nCount_RNA <1000, or nCount_RNA >20000 were removed from the dataset prior to analysis. 10X analysis was performed using Seurat 5.1.0^82^ where cells were visualized using the uniform manifold approximation and projection (UMAP) algorithm. The number of dimensions used with RunPCA, RunUMAP, and FindNeighbors was determined using JackStraw with a *p* value cutoff of 0.05. Clusters were determined via FindClusters using the leiden algorithm. To subset eye cells from all other cells in the dataset, *ovo*+ clusters 117, 99, and 127 were selected along with any cells with normalized *ovo* (dd_48430) expression above 0.5. Within the clustered eye subset, the post-mitotic progenitor cluster was further divided into three categories: optic cup post-mitotic progenitors expressing either *sp6/9* (dd_17385), *dlx* (dd_19040), *tyrosinase* (dd_34399) or *tph* (dd_8392) and not expressing *otxA* (dd_14633) or *FoxQD* (dd_50245), photoreceptor post-mitotic progenitors expressing either *otxA* or *FoxQD* but not expressing *sp6/9, dlx, tph,* or *tyrosinase*, and unspecified post-mitotic progenitors that fit neither criteria; all expression cutoffs used normalized expression values of 0.1, except for *otxA* that was 1. To subset all anterior and posterior X1 cells to neoblasts, cells with normalized *smedwi-*1 (dd_659) expression above 0.5 were selected.^30^ To subset all anterior and posterior X2 cells to post-mitotic progenitors, cells with normalized *smedwi-1* expression below 3.5 and *p4hb* (dd_250) expression above 1.5 were selected.^30^ UMAP plots of gene expression were created using Seurat’s FeaturePlot function with order = T. Marker genes were identified with Seurat’s FindAllMarkers function with only.pos = T and filtered by those with p_val_adj <0.05. Using average expression values obtained from Seurat’s PseudobulkExpression function, all heatmaps were constructed by scaling data by genes using R’s base scale() function with center = F and plotting via ComplexHeatmap 2.14.0.^69^ Violin plots and QC stats for all lanes were created using Seurat’s VlnPlot function. Dot plots were created using Seurat’s DotPlot function with scale = F. The dot plot of discoidin genes across cell types was created using published data.^83^ Blast annotations for tables were created by performing a translated blast (blastx) of the complete dd_Smed_v6 transcriptome to human (GRCh38.p13), mouse (GRCm39), and fly (dm6) proteomes and filtering hits to those with an E-value below 0.05. SignalP and Pfam annotations were created by providing the largest ORF for each Smed_v6 transcript, determined via ORFFinder 1.8 (https://github.com/Chokyotager/ORFFinder/), to signalP6^75^ using the Pfam-A.hmm model from Pfam 37.3.^76^ GO term annotations were taken from PlanMine.^66^ Digiworm annotations were extracted^83^ from digiworm.wi.mit.edu and Smed_v4 transcripts were paired with corresponding Smed_v6 transcripts. Because the GTF file used for mapping reduces isoforms to a single gene, the isoform that contained the most complete set of annotations and was present in the GTF file was used for annotations. Venn diagrams were created in R using the VennDiagram package. For the Venn diagram of all genes expressed in the eye, genes expressed in progenitors, optic cup cells, or photoreceptors were determined by first finding all genes with an average expression above 0.06 in any of the three groups using Seurat’s PseudobulkExpression and then by flagging a gene as expressed in each of the three groups if its scaled expression using R’s base scale() function with center = F was greater than 0.5. For additional Venn diagrams, the resulting list was filtered either by genes included in the RNAi screen or by genes from the RNAi screen that resulted in a discernible phenotype. Scatterplots and pseudotime plots were created using ggplot2 3.5.1. Pseudotime gene expression plots used normalized gene expression values smoothed across pseudotime via mgcv’s gam function and then scaled from 0 to 1. The correlation plot of eye cell clusters was created via ComplexHeatmap following Psych’s corr.test on average gene expression for each cluster obtained via Seurat’s PseudobulkExpression with features restricted only to genes shown in the pseudotime gene expression plots and normalized per gene across clusters. Binary expression heatmaps were not scaled and used an expression cutoff of 0.1. For manipulation of data, including for results from URD and DESeq2 mentioned below, dplyr 1.1.4 and tidyr 1.3.1 were used (https://dplyr.tidyverse.org).

#### URD analyses

URD analysis was performed using URD 1.1.1.^31^ Using eye cells only, eye neoblasts were specified as the root stage and optic cup cells and photoreceptors as two tip stages. PCA values for the URD object and variable features for tip stages were imported from Seurat analyses. Steps during trajectory analysis were performed with the following parameters: createURD with min.genes = 0 because filtering was already performed via Seurat, calcDM with knn = 100 and sigma = 16, floodPseudotime with *n* = 50, calcPCA for tips with mp.factor = 3 and graphClustering for tips with num.nn = 400, pseudotimeDetermineLogistic with optimal.cells.forward = 20 and max.cells.back = 40, simulateRandomWalksFromTips with n.per.tip = 25000 and max.steps = 5000, and buildTree with divergence.method = “preference”, cells.per.pseudotime.bin = 25, bins.per.pseudotime.window = 8, save.all.breakpoint.info = T, p.thresh = 0.001. URD tree plots were created using URD’s plotTree function.

#### Protein domain analyses

The longest predicted ORF of DDR family members from the dd_v6 transcriptome assembly was inspected for domain architecture similarities using SMART with HMMER searches of Outlier homologs, PFAM domains, and signal peptide prediction. Distant homology search was performed using HHpred from MPI Bioinformatics Toolkit with default parameter settings.^84^ Homology search for dd_9381 was performed using blastp against the non-redundant protein sequences database with an E-value threshold of 0.05. The predicted protein structures were generated by AlphaFold 3 through AlphaFold Server.^85^ Structures of individual protein domains are extracted manually and compared to PDB25 database using DALI.^86^ DeepTMHMM-1.0 is used for predicting protein transmembrane regions.^87^

#### Gene cloning

All constructs for the RNAi screen were cloned from cDNA into the pGEM vector (Promega). These constructs were used to synthesize RNA probes and double-stranded RNA (dsRNA) for RNAi experiments. All genes used are identified with a Smed_v6_dd contig id that can be found online at https://planmine.mpinat.mpg.de/planmine/begin.do.

#### Double-stranded RNA synthesis for RNAi

dsRNA was prepared from *in vitro* transcription reactions (Promega) using PCR-generated forward and reverse templates with flanking T7 promoters (TAATACGACTCACTATAGGG). Each template (32 μL) was mixed with 3.2 μL of 100 mM rNTPs (Promega); 0.012 μL of 100 μM dithiothreitol (DTT; Promega); 8 μL of T7 polymerase; and 48 μL of 5x Transcription optimized buffer (Promega). Reactions were incubated overnight at 37°C. Forward and reverse strands were combined and RNA was purified by ethanol precipitation, and re-suspended in a final volume of 25 μL milliQ H2O. dsRNA was heated at 95°C for 5 min followed by cooling to RT. Animals were starved for 1–2 weeks prior to first RNAi feeding and were fed twice a week. RNAi food mixture was prepared using 25 μL dsRNA and 50 μL planarian food (homogenized beef liver). *C. elegans unc-22* was used as the control condition. All RNAi experiments that show a phenotype have been independently repeated at least three times. Phenotype details with quantification can be found in Table S3.

#### Eye cell dissociations and eye transplantations

For all surgical procedures, animals were placed on moist filter paper on a cold Peltier block to limit movement. Eye transplants were performed as described before.^6^ Briefly, eyes from wild-type or RNAi condition animals were surgically removed and transplanted into wild-type animals. Transplanted animals were then immobilized using Type IV, 5% ultra-low melting agarose (Sigma). The solidified gel was then covered with filter WhatmanTM paper (GE Healthcare, Life Sciences) and was soaked in Holtfreter’s Solution. Animals were left at 10°C overnight, and were rescued the following day by cutting the surrounding gel and transferring them into planarian water.

For eye cell dissociations for DIC imaging experiments, planarian head pieces containing eyes were amputated for gentle dissociation using a surgical scalpel and were incubated in Trypsin at RT for 5–10 min. The pieces were gently pipetted to isolate complete pure eyes into CMF (RT). Single eyes were placed on a microscopy slide in 20–30μL CMF, and were covered with a coverslip to gently squash the overall structure to reveal rhabdomeres. Images were taken using a Zeiss Axio Imager and Axiovision software.

#### TEM

Animals were fixed in a 2.5% glutaraldehyde and 2.5% formaldehyde in 0.1M sodium cacodylate buffer (pH 7.4, Electron Microscopy Sciences) for 2 h at RT. Small pieces (1–2 mm cubes containing the eyes) of fixed tissue were washed in 0.1M cacodylate buffer and postfixed with 1% osmium tetroxide (OsO4)/1.5% potassium ferrocyanide (KFeCN6) for 1 h, washed in water 2x, 1x in 50mM maleate buffer pH 5.15 (MB) and incubated in 1% uranyl acetate in MB for 1 h followed by 1x wash in MB, 2x in water and subsequent dehydration in grades of alcohol (10 min each; 50%, 70%, 90%, 2 × 10 min 100%). The samples were then put in propylene oxide for 1 h and infiltrated ON in a 1:1 mixture of propylene oxide and TAAB Epon (TAAB Laboratories Equipment Ltd, https://taab.co.uk). The following day the samples were embedded in TAAB Epon and polymerized at 60 C for 48 h. Ultrathin sections (80 nm) were cut on a Reichert Ultracut-S microtome, picked up onto copper grids, stained with lead citrate and examined in a JEOL1200EX TEM and images were recorded with an AMT 2k CCD camera. All TEM images shown in this study are representative of at least three animals in each of two independent RNAi experiments.

#### Whole-mount fluorescent *in situ* hybridizations

Animals were killed in 5% NAC in PBS for 5 min before fixation in 4% formaldehyde for 20 min. Fixative was removed and worms were rinsed 2X with PBSTx (PBS +0.1% Triton X-100). Animals were dehydrated and stored in methanol at −20°C. RNA probes were synthesized as described previously.^88^ Fixed animals were bleached, rehydrated and treated with proteinase K (1 μg/mL) in 1xPBSTx. Following overnight hybridizations, samples were washed twice in pre-hyb solution, 1:1 pre-hyb-2X SSC, 2X SSC, 0.2X SSC, PBSTx. Subsequently, blocking was performed in 10% Roche Western Blocking reagent in PBSTx. Animals were incubated in anti-DIG or anti-FITC antibody overnight at 4°C in the blocking solution. Six post-antibody washes with PBST were performed (10 min each at RT) and animals were incubated in a rhodamine tyramide solution for 10 min RT. Peroxidase inactivation with 1% sodium azide was done for 90 min at RT. Another six PBSTx washes were performed following tyramide labeling or inactivation and samples were then incubated overnight at 4°C in a solution containing 1:7500 dilution of the VC-1 antibody (in PBST with 0.1% BSA). Six post-antibody washes with PBST were performed (10 min each at RT) and animals were incubated in a secondary antibody solution in PBST with 10% Horse Serum (1:500 dilution, anti-mouse Alexa 488). Specimens were counterstained with DAPI overnight (Sigma, 1 μg/mL in PBSTx).

#### Photoablation

Planarians were depigmented through continuous exposure to 5000 lux of red LED light (ABI) in a temperature-controlled chamber for a period of 7 days. Light intensity was verified using a digital light meter (LX1010B, Dr. Meter). After depigmentation, animals were fixed as previously described.

#### EdU delivery and labeling

Animals were soaked in 1.25 mg/mL of EdU (Vector Laboratories, CCT-1403–100) in planarian water overnight at RT. Controls were treated with equivalent DMSO concentration. Animals were then incubated in 5g/L Instant Ocean until fixation. Animals were then fixed as previously described. Animals were processed using the FISH protocol with a modified EdU labeling step before probe hybridization. Animals were incubated in the dark for 30 min in an azide click reaction containing 1% 100 mM CuSO_4_, 0.1% 10mM azide-fluorophore 545 (Sigma, 760757), 20% 50 mM ascorbic acid from (+) sodium-L-ascorbate in PBS. Ascorbic acid was made fresh for each reaction. After EdU labeling animals were washed 6X in PBSTx and FISH protocol was continued.

#### Microscopy and image analysis

Fluorescent images were taken with a Leica Stellaris Confocal Microscope. All images are maximum intensity projections, except the ones showing eye morphological defects in Figure 4 and Figure S4 that are single confocal plane images at the mid DV point of the eye. Images were processed using ImageJ (Fiji). Brightfield images were taken with a Zeiss Discovery Microscope. Cell counting was performed manually after blinding control and experimental conditions. All images shown are anterior and dorsal up. Animals used in each condition are shown in the figure panels.

#### RNA sequencing and proteomics analyses

For bulk RNA sequencing, *DDR-1* and control RNAi heads were surgically isolated, incubated in Trizol and mRNA purified following the manufacture instructions. Five head pieces were pooled in the same sample. Five independent replicates were used per condition. Sample libraries were prepared by the WIGTC core (Whitehead Institute for Biomedical Research, Genome Technology Core) using the Swift Rapid mRNA-Seq Kit, barcoded with SwiftRNA dual-indexed adapters (IDT DNA Technologies). Libraries were sequenced on a NovaSeq 6000 with 150 ×150 bp reads. Reads were mapped to the dd_Smed_v6 transcriptome with Kallisto^77^ and isoforms of the same gene were collapsed by summing counts. Differential expression analysis was performed using DESeq2.^89^ Using gene counts per sample, heatmaps were generated as previously described for single cell gene expression data. Expression is considered significantly downregulated if both adjust *p* value <0.05 and log2FC < −0.5. In the table of results, genes were considered expressed in single cell sequencing data if the average.

For the proteomics analysis, eyes were isolated from *DDR-1* and control RNA animals as described for single cell sequencing above. 20 eyes were pooled in each sample, and four independent replicates were used in each condition. Eyes were incubated in 5% SDS in 50mM TEAB, proteins reduced at 55°C for 15 min with 10mM TCEP, and alkylated at RT for 15 min with 40mM IAA. Proteins were extracted via the SP3 method as in.^90^ Proteins were bound to SP3 beads by adding a 4X volume of 100% (v/v) ethanol followed by three washing steps with 200 μL 80% (v/v) ethanol in LC-MS grade water. Proteins were proteolytically digested overnight in a shaking incubator at 37°C at 115 RPM with trypsin/LysC mix (1:100) in 50mM TEAB. The following day, an additional dose of trypsin/LysC mix was added (1:100) in 50mM TEAB and the digestion proceeded for 4 h at 37°C. The peptide digests were purified using Stage Tips following the protocol from.^91^ The peptides were then dried using a Speed-Vac concentrator and reconstituted in 0.2% (v/v) formic acid in MS-grade water for LC-MS analysis.

Mass spectrometry was performed using an Orbitrap Exploris mass spectrometer equipped with a FAIMS Pro interface connected to an Easy-nLC 1200 chromatography system, all from Thermo Fisher Scientific (Waltham, MA, USA). NanoLC separation utilized an Acclaim PepMap trap column (75 μm × 2 cm) combined with an EasySpray ES902 column (75 μm × 25 cm, 100 A) ^_^ from Thermo Fisher Scientific. Peptide extracts were injected with a volume of 5 μL. Peptide separation was conducted with a mobile phase consisting of 0.1% (v/v) formic acid in water (solution A) and 0.1% (v/v) formic acid in 80% (v/v) acetonitrile (solution B), flowing at 300 nL/min, while the column temperature was held constant at 30°C. Peptides were separated on a gradient of 1–25% B for 90 min, 25–40% B for 30 min, 40–95% B for 10 min, 95% B over 10 min 95–2% B for 2 min, 2% B over 2 min, 2–95% B for 2 min, 95% B over 2 min, 95–2% B for 2 min, and 2% B over 2 min. Using the MS in positive mode, the ion source temperature was set to 270°C, and ionized peptides were passed through the FAIMS Pro unit at −50 V. Mass spectra were collected in MS1 mode with a resolution of 120,000, spanning the mass range of 400–1000 m/z. This was carried out using custom automatic gain control (AGC = 300) settings and automatic injection time. For MS2 data collection, the mass spectrometer was operated in DIA mode with a resolution of 30,000. MS2 spectra were gathered across a precursor mass range of m/z 400–1000, utilizing isolation windows of m/z 20, with no overlaps, a standard AGC target, and 30% normalized collision energy.

Proteomic data were analyzed using DIA-NN version 1.8.^92^ Raw LC-MS files were processed in library-free mode with in silico digestion of a custom *Schmidtea mediterranea* FASTA database. Search parameters included Trypsin/P specificity allowing up to one missed cleavage and N-terminal methionine excision. Fixed modification of cysteine residues (carbamidomethylation) was applied. Peptides were restricted to 7–30 amino acids, with precursor charges of 1–4 and m/z ranges of 300–1800 for precursors and 200–1800 for fragments. The precursor-level false discovery rate (FDR) was set to 1.0%. DIA-NN was run with isotopologue detection, heuristic protein interference, exclusion of shared spectra, and gene-level protein inference. The neural network classifier operated in single-pass mode. Quantification used the robust LC (high precision) strategy with RT-dependent cross-run normalization. Smart profiling was employed for spectral library generation. Performance was optimized for speed and memory efficiency.

#### Behavior assays

Behavior assays were performed as described.^6^ Briefly, a gradient layout for the behavior arena was generated using Adobe Illustrator CC software. The gradient arena was displayed on a horizontally placed iPad continuously. A rectangular one-well plate containing planarian water was placed on top of the iPad and the arena was covered with a box to eliminate any directional light from the test environment. An iPhone was placed on top of the box to record videos of the behaving animals. Animals were placed in positions 5 and 6 of the arena at the start of each trial. Positions of each animal at the end of each minute were recorded for a total of 5 min. Time 0 values were not analyzed for significant deviation from random distribution.

### QUANTIFICATION AND STATISTICAL ANALYSIS

Statistical analyses were performed using the Prism software package (GraphPad Inc., La Jolla, CA). Comparisons between the means of two populations were done by a Student’s *t* test. Comparisons of means between multiple populations were done by one-way ANOVA. For behavioral analyses, one-sample *t* test comparing each column mean with a hypothetical value of 6 corresponding to chance (*n* ~10 animals per cohort) was used. Bonferroni correction was applied. Significance was defined as *p* < 0.05.

## Supplementary Material

1

2

3

4

5

6

7

8

Supplemental information can be found online at https://doi.org/10.1016/j.celrep.2026.117245.

## Figures and Tables

**KEY RESOURCES TABLE:** 

REAGENT or RESOURCE	SOURCE	IDENTIFIER
Antibodies		
anti-Digoxigenin-POD	Roche	Cat# 11 207 733 910; RRID: AB_514500
anti-mouse-Alexa 488	Life Technologies	Cat# A11001
anti-arrestin Antibody (mouse)	Agata Lab	N/A
Anti-muscle 6G10 (mouse)	DSHB	http://dshb.biology.uiowa.edu; RRID: AB_2619613
EdU	Vector Laboratories	CCT-1403-100
azide-fluorophore 545	Sigma Aldrich	Cat# 760757
Bacterial and virus strains		
NEB^®^ 10-beta Competent E. coli (High Efficiency)	NEB	Cat# C3019P
Critical commercial assays		
Swift Rapid RNA Library kit	IDT DNA Technologies	Cat# R2096
Chemicals, peptides, and recombinant proteins		
BSA	Sigma Aldrich	Cat# A8806
Collagenase	Sigma Aldrich	Cat# C0130-500MG
Trypsin	Gibco, Thermo-Fisher Scientific	Cat# 25200-056
Western Blocking Reagent	Roche Diagnostics	Cat# WESTBL-RO
N-acetylcysteine	Sigma Aldrich	Cat# A7250
Formaldehyde	Fisher Scientific	Cat# F79500
Papain	Worthington Biochemical Corporation	Cat# LK003150
DPBS with Calcium and Magnesium	Gibco, Thermo-Fisher Scientific	Cat# 14040117
DPBS no Calcium or Magnesium	Gibco, Thermo-Fisher Scientific	Cat# 14190250
B-27^™^ Plus Supplement	Gibco, Thermo-Fisher Scientific	Cat# A3582801
Form/Glut 2.5% in 0.1M Sodium Cacodylate Buffer	Electron Microscopy Sciences	SKU: 15949
Horse serum	HyClone	SH30074
Propridium iodide (PI)	Sigma Aldrich	Cat# P4864
Hoechst	Life Technologies	Cat# H3570
EdU	Vector Laboratories	CCT-1403-100
azide-fluorophore 545	Sigma Aldrich	Cat# 760757
Deposited data		
Dresden transcriptome v6	Rozanski et al.^66^	http://planmine.mpi-cbg.de/planmine/begin.do
10X eye scRNA-seq	This paper	SRA: PRJNA1269575
10X neoblasts and post-mitotic progenitors scRNA-seq	King et al.^30^	SRA: PRJNA1067154
Experimental models: Organisms/strains		
Asexual Schimdtea mediterranea strain ClW4	Reddien lab	N/A
Oligonucleotides		
Please find them in Table S3	N/A	N/A
Recombinant DNA		
pGEM-T easy vector system	Promega	Cat# A1360
Software and algorithms		
Seurat package	Satija et al.^67^	https://satijalab.org/seurat
scDblFinder	Germain et al.^68^	github.com/plger/scDblFinder
ComplexHeatmap	Gu et al.^69^	github.com/jokergoo/ComplexHeatmap
URD	Farrell et al.^31^	github.com/farrellja/URD
ggplot2	Wickham^70^	ggplot2.tidyverse.org
ggrepel	Slowikowski^71^	ggrepel.slowkow.com
dplyr	Wickham et al.^72^	dplyr.tidyverse.org
tibble	Müller & Wickham^73^	tibble.tidyverse.org
tidyr	Wickham et al.^72^	tidyr.tidyverse.org
VennDiagram	Chen & Boutros^74^	github.com/uclahs-cds/package-VennDiagram
ORFFinder	Github: Chokyotager	github.com/Chokyotager/ORFFinder
SignalP	Teufel et al.^75^	services.healthtech.dtu.dk/services/SignalP-6.0
Pfam	Mistry et al.^76^	pfam.xfam.org
Kallisto	Bray et al.^77^	github.com/pachterlab/kallisto
10X Genomics Cell Ranger	Zheng et al.^78^	10xgenomics.com/support/software/cell-ranger/7.2
FIJI	ImageJ	https://imagej.net/Fiji
ZEN digital imaging software	Zeiss	https://www.zeiss.com/microscopy/us/ products/microscope-software/zen.html
GraphPad Prism	GraphPad Software	https://www.graphpad.com/scientific-software/prism/
AlphaFold 3	AlphaFold Server	https://alphafoldserver.com/
SMART	SMART Server	https://smart.embl.de/smart/change_mode.cgi

## Highlights

- scRNA-seq and RNAi screening identify genes controlling eye regeneration steps
- Seven genes affect specification, differentiation, and/or maturation of eye progenitors
- 145 genes are involved in planarian optic cup and rhabdomere formation
- DDR genes promote PR projections, progenitor trapping, and transparency
