# Supplementary material for: Single-cell transcriptomics and RNAi screening define a hierarchical program of planarian eye regeneration
Source: Cell Rep. Author manuscript; Available in PMC 2026 Jul 1. (PMC13322009; doi:10.1016/j.celrep.2026.117245)
Supplement: 1 [file NIHMS2170797-supplement-1.pdf]

Cell Reports, Volume *45*

Supplemental information

Single-cell transcriptomics and RNAi screening define a hierarchical program of planarian eye regeneration

M. Lucila Scimone, Bryanna I. Canales, Patrick Aoude, Kutay D. Atabay, Cyrille W. Teforlack, and Peter W. Reddien

Supplemental Information

Document S1. Figures S1-S7

Figure S1. Planarian eye regeneration and single eye cell transcriptomics. (A) FISH and immunostaining show an eye regeneration timecourse following decapitation. Dorsal projections (yellow arrows) are apparent between 10 and 15 dpa. (B) Graph shows OC and PR numbers (mean ± SD) during regeneration following head amputation. (C) FISH and immunostaining show an eye regeneration timecourse following unilateral eye resection. (D) Graph shows OC and PR numbers (mean ± SD) during regeneration following unilateral eye resection. (E) Quality controls of the single cell sequencing dataset. (F) Total cell numbers and mean counts per lane. (G) UMAP plot shows a total of 133 cell clusters. (H) UMAP plots show *smedwi-1* and eye markers expression in the total single cell population. (I) UMAP plots show cell lane origin and eye markers expression in the *ovo*+ cells subclustering. (J) Venn diagrams show eye cell type distribution of all genes expressed in the eye and all genes RNAi. (K) Schematics of the surgeries utilized in the RNAi screen. (L) FISH and immunostaining show eye expression of candidate genes validating the single cell sequencing dataset. Scale bars, 50 μm.

Figure S2. Gene expression in *ovo*+ cells and eye resection phenotypes following RNAi. (A) UMAP plots show gene expression of neoblast and post-mitotic progenitor markers in the *ovo*+ cells. (B) FISH and immunostaining show regeneration defects following unilateral eye resection and RNAi. (C) Coexpression of *sp6/9* and *otxA* in *ovo*+ progenitor cells. Cartoon shows region of images displayed. Scale bars, 50 μm.

Figure S3. Genes required for PR differentiation and OC maturation. (A) Gcr591 and Rgs-1 protein structure and 3D models generated with AlphaFold. (B) Graph shows a subset of genes (red dots) expressed in eye progenitors but not in differentiated cells. (C) Violin plot (left) shows expression of eye markers along eye differentiation trajectories. Dot plot (middle) shows top 10 markers enrich in each differentiation cell subset. Heatmap (right) shows correlation coefficients between progenitor populations and differentiated cells. (D) UMAP plots (left) and URD analyses (right) show expression in *ovo*+ cells of genes required for eye cell differentiation. (E) Line graphs show gene expression during the PR differentiation trajectory. (F) Dot plots show expression of eye differentiation genes in all neoblast and post-mitotic progenitor (pmP) clusters. UMAP plots show all clusters in the neoblast compartment (top) and the pmP (bottom). (G) FISH and immunostainings show eye regeneration defects following RNAi. (H) FISH and

immunostainings show normal eye morphology in *rgs-1* and *gcr591* RNAi animals but absence of PRs following *klf* RNAi. (I) TEM shows PR absence (rh, rhabdomeres; oc, optic cup) in a *klf* RNAi animal. (J) Live images show loss of OC melanin in uninjured RNAi animals. (K) 3D protein model of Melanoir generated with AlphaFold. (L) UMAP plots show OC expression of genes required for melanin formation. (M) Line graphs show gene expression during the OC differentiation trajectory. (N) FISH and immunostainings show expression of different OC cell markers in RNAi animals without OC melanin. (O) Behavior analyses show negative phototaxis defects in RNAi animals without OC melanin. Scale bars, 20 μm.

Figure S4. Genes required for rhabdomere formation and eye morphogenesis. (A) DIC images showing rhabdomere structure in a wild-type eye prep over time, rhabdomeres, rh. (B) FISH and immunostainings show rhabdomere regeneration timecourse in wild-type animals. (C) FISH and immunostainings show examples of rhabdomere regeneration defects following RNAi at 7 dpa. (D) Behavior analyses show defects in negative phototaxis in RNAi animals that regenerate very short or no rhabdomeres. (E) Immunostaining shows optic chiasma formation in animals that did not regenerate rhabdomeres following RNAi. (F) UMAP plots show expression of genes required for rhabdomere regeneration in the *ovo*+ cells (left). Protein structure of those genes (right). (G) FISH and immunostaining analyses show eye morphogenesis defects following RNAi. (H) Heatmap shows expression through eye cell differentiation of all genes that caused eye morphogenesis defects following RNAi. (I) Behavior analyses show negative phototaxis defects in RNAi animals with eye morphogenesis defects. Scale bars, 50 μm.

Figure S5. Formation of the eye transparent region. (A) Live images show decreased TR in an uninjured *klf* RNAi animal. (B) Tracing of dorsal projections (left) and immunostaining showing dorsal projections towards the epidermis (DAPI) in a regenerating *sp6/9* RNAi animal. (C) FISH and immunostaining show normal eye morphology in a partially depigmented animal. (D) Live images following eye transplantations show eye-intrinsic capability to generate the TR. Cartoons (top) summarize the procedure. (E) Dot plots show expression of matrisome genes in different eye differentiation subsets. (F) Live images show no defect in the maintenance of the eye TR following RNAi of collagen genes. (G) Schematics of the bulk-sequencing experimental design (left). Heatmap showing significantly downregulated genes following *DDR-1* RNAi. (H) Schematics of the proteomics experimental design (left). Heatmap showing significantly downregulated proteins following *DDR-1* RNAi. (I,J) FISH and immunostaining show eye regeneration defects following *DDR-1* RNAi: ectopic dorsal projection bundling and disorganization (I) and loss of eye TR (J). (K) Immunostaining shows normal optic chiasma

formation in a *slc8a-1* RNAi animal with reduced/short PR dorsal projections. (L) Behavior analyses show negative phototaxis defects in *slc8a-1* RNAi animals with reduced PR dorsal projections. Cartoons show regions of images displayed. Scale bars, 50 μm.

Figure S6. Genes required for eye progenitor trapping. (A) FISH and immunostaining show progenitor trapping defects in a *klf* and *otxA* RNAi animals (yellow arrows point to differentiated ectopic OC cells) following unilateral eye resection in head fragments. (B) Protein structure and 3D models generated with AlphaFold of genes involved in eye progenitor trapping. (C) UMAP plots show expression of genes required for eye progenitor trapping. (D) FISH and immunostaining show defects in eye progenitor trapping following unilateral eye resection in morphallaxing head fragments following RNAi (left). UMAP plots (right) showing expression of those genes in *ovo*+ cells. Heatmap (bottom) shows expression of eye progenitor trapping genes along the eye differentiation trajectory. (E) Live images of uninjured RNAi animals show no defect in the location of the eye nucleation following eye resection, indicating no shifts in the target zone. Cartoons show regions of images displayed. Scale bars, 50 μm.

Figure S7. DDR genes are required for eye TR formation, dendritic arborization and eye progenitor trapping. (A). Live image shows no defect in eye regeneration location following unilateral eye resection in an uninjured *DDR-1* RNAi animal indicating no target zone shifts. (B) FISH and immunostaining show ectopic anterior eyes (yellow arrows) in a *DDR-1* RNAi morphallaxing head fragment showing eye progenitor trapping defects. (C) Live image shows no defect in eye regeneration location following unilateral eye resection in an uninjured *slc8a-1* RNAi animal indicating no target zone shifts. (D) Protein domain structure of DDR family members. Dot plots show DDR gene expression in different cell types (left) and in different eye differentiation subsets (right). Heatmap shows *DDR* gene expression through the PR and OC differentiation trajectories. (E) Live images show no defect in eye regeneration location following unilateral eye resection in an uninjured *tDDR-2* or *DDR-3* RNAi animal indicating no target zone shifts. (F) FISH and immunostaining show mild eye regeneration defects following *DDR-2* RNAi, and no defects after *tDDR-3* RNAi. (G) FISH and immunostaining show no eye trapping defects following *DDR-2* or *tDDR-3* RNAi. Cartoons show regions of images displayed. Scale bars, 50 μm.

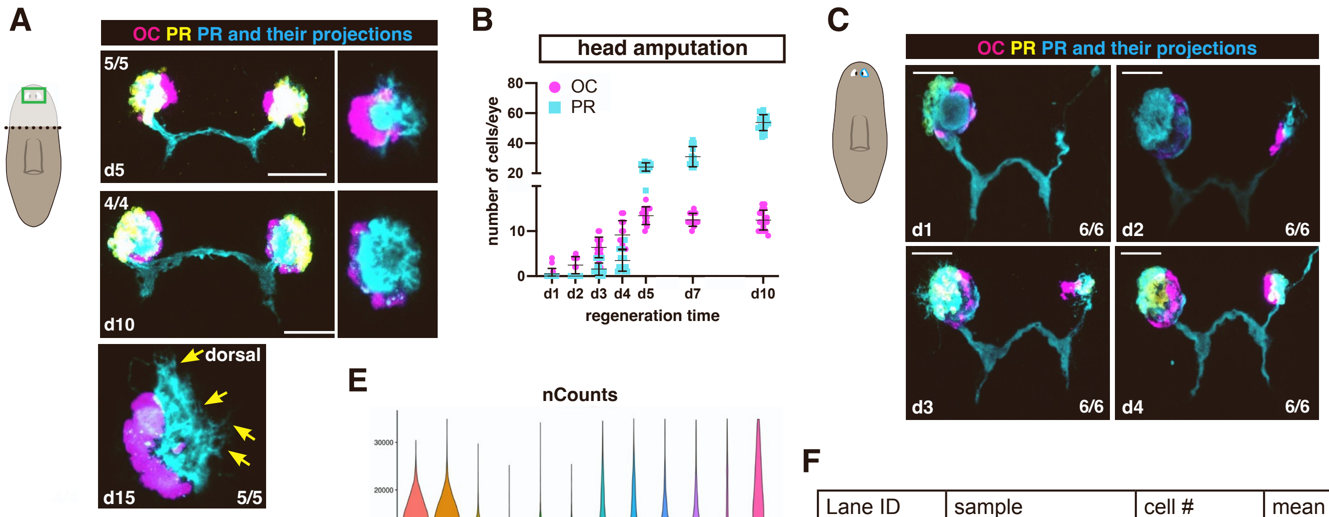

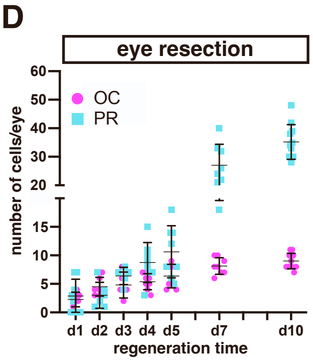

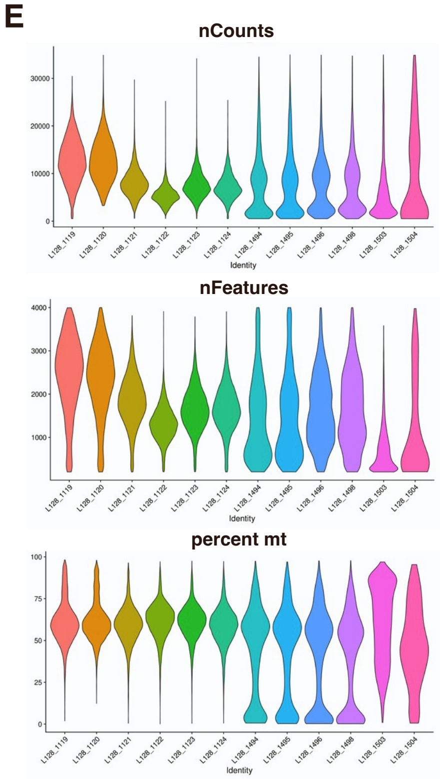

F

Lane ID	sample	cell #	mean counts/ cell
L128_1119	Anterior X1 *	6849	12749
L128_1120	Posterior X1 *	7306	12759
L128_1121	Anterior X2 A *	7672	8094
L128_1122	Anterior X2 B *	7647	5422
L128_1123	Posterior X2 A *	7181	7644
L128_1124	Posterior X2 B *	7458	7236
L128_1494	3 dpa A	4775	7771
L128_1495	3 dpa B	4680	7954
L128_1496	5 dpa X2/Xins A	4728	7780
L128_1498	5 dpa X2/Xins B	4451	7642
L128_1503	intact eyes	9278	6455
L128_1504	extra pure intact eyes	246	9708

* From King et al.

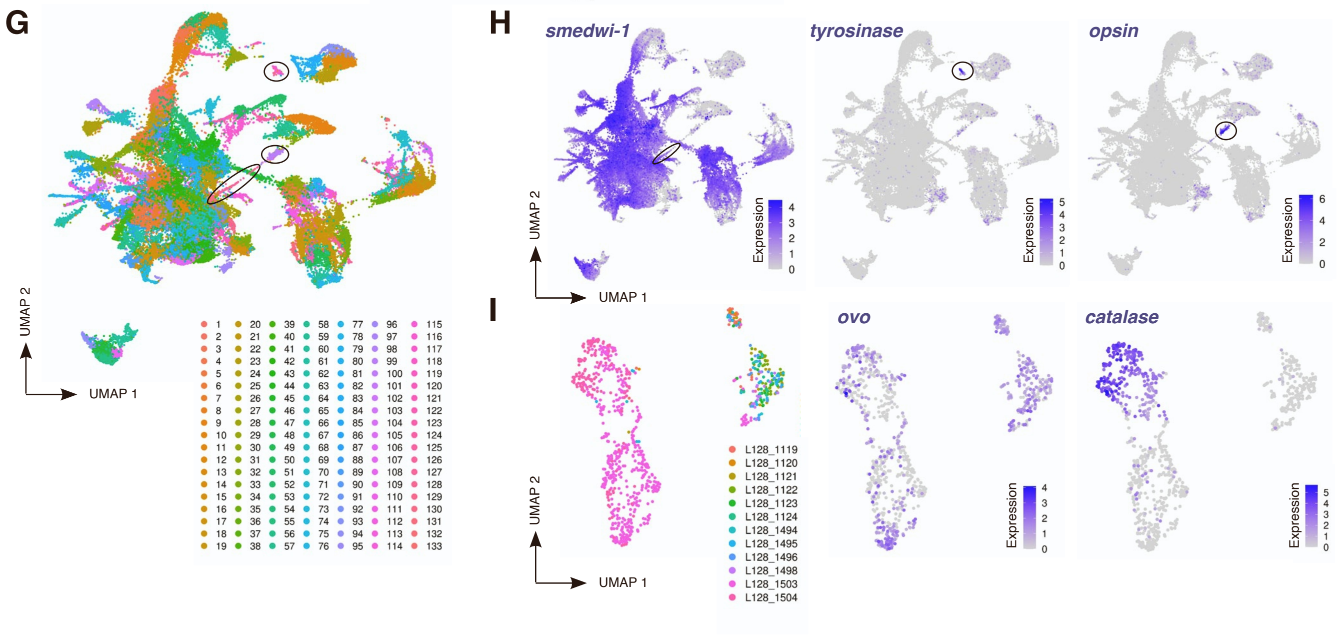

Figure S1

J

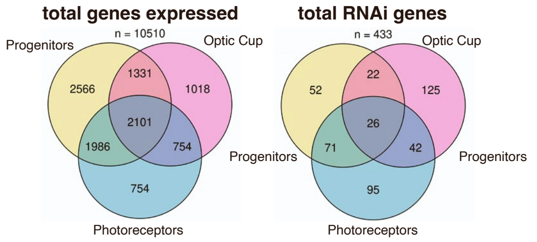

K

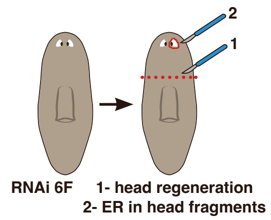

L

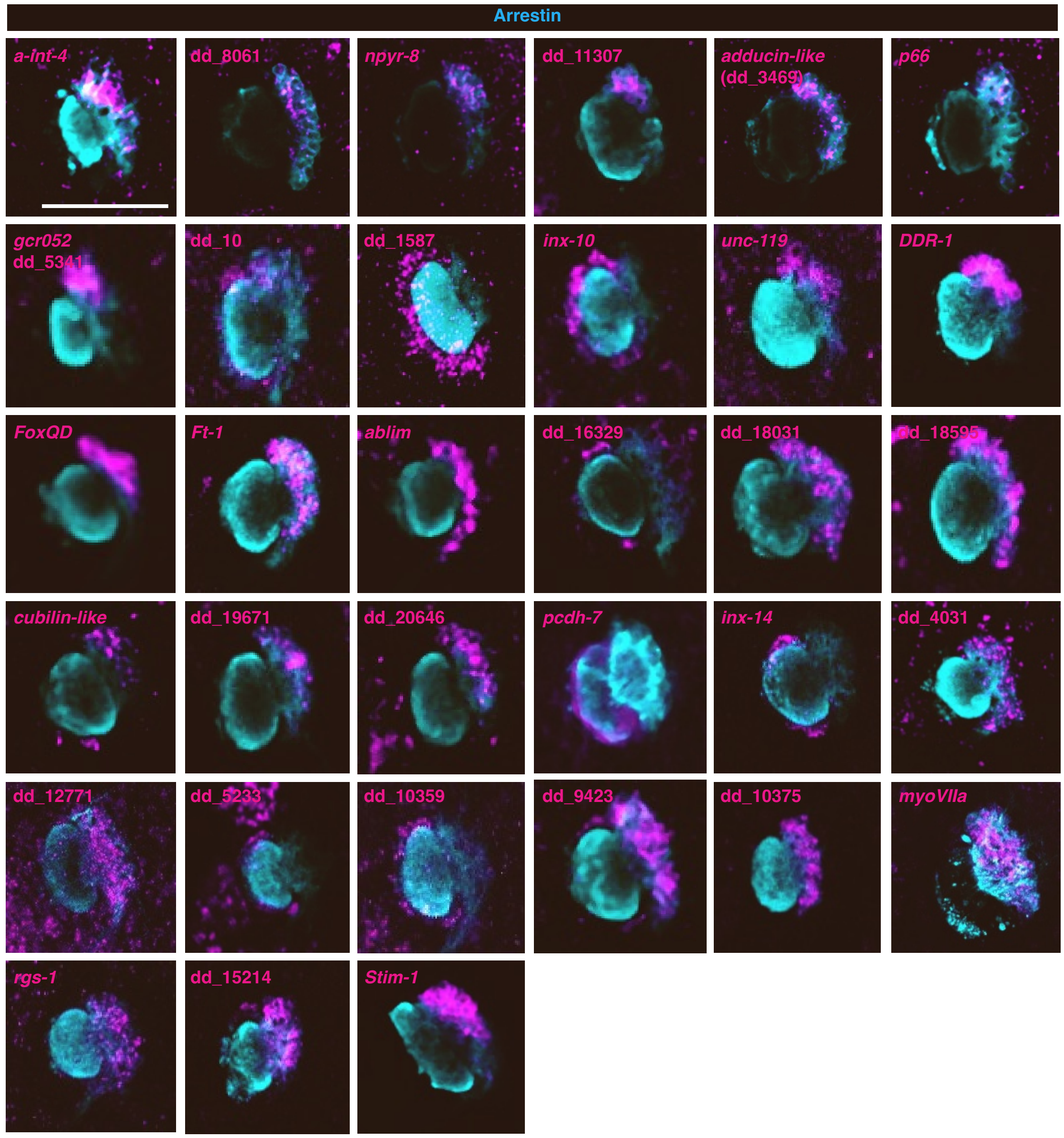

Figure S1

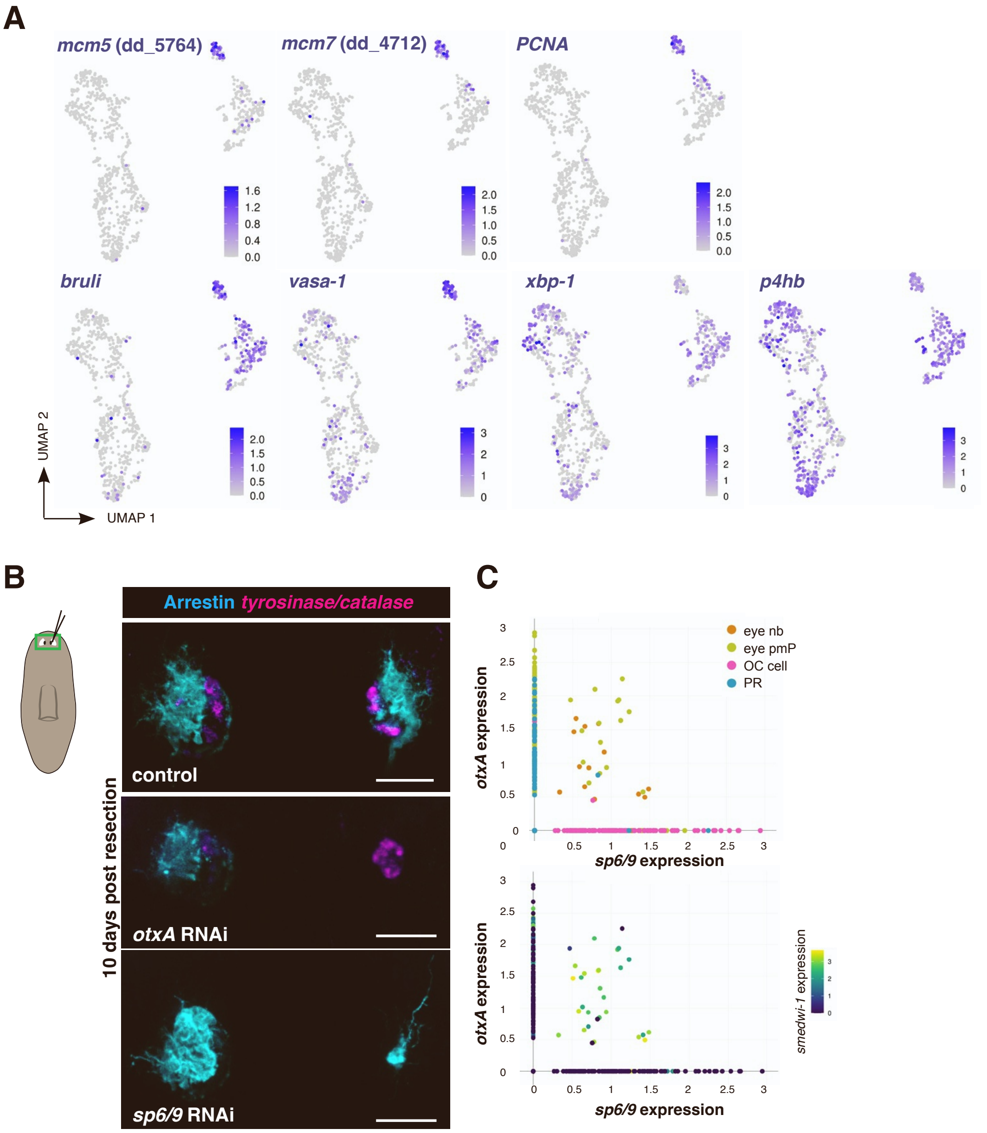
A
mcm5 (dd_5764)
mcm7 (dd_4712)
PCNA
bruli
vasa-1
xbp-1
p4hb
UMAP 2
UMAP 1
B
Arrestin tyrosinase/catalase
control
otxA RNAi
sp6/9 RNAi
10 days post resection
C
eye nb
eye pmP
OC cell
PR
otxA expression
sp6/9 expression
smedwi-1 expression

Figure S2

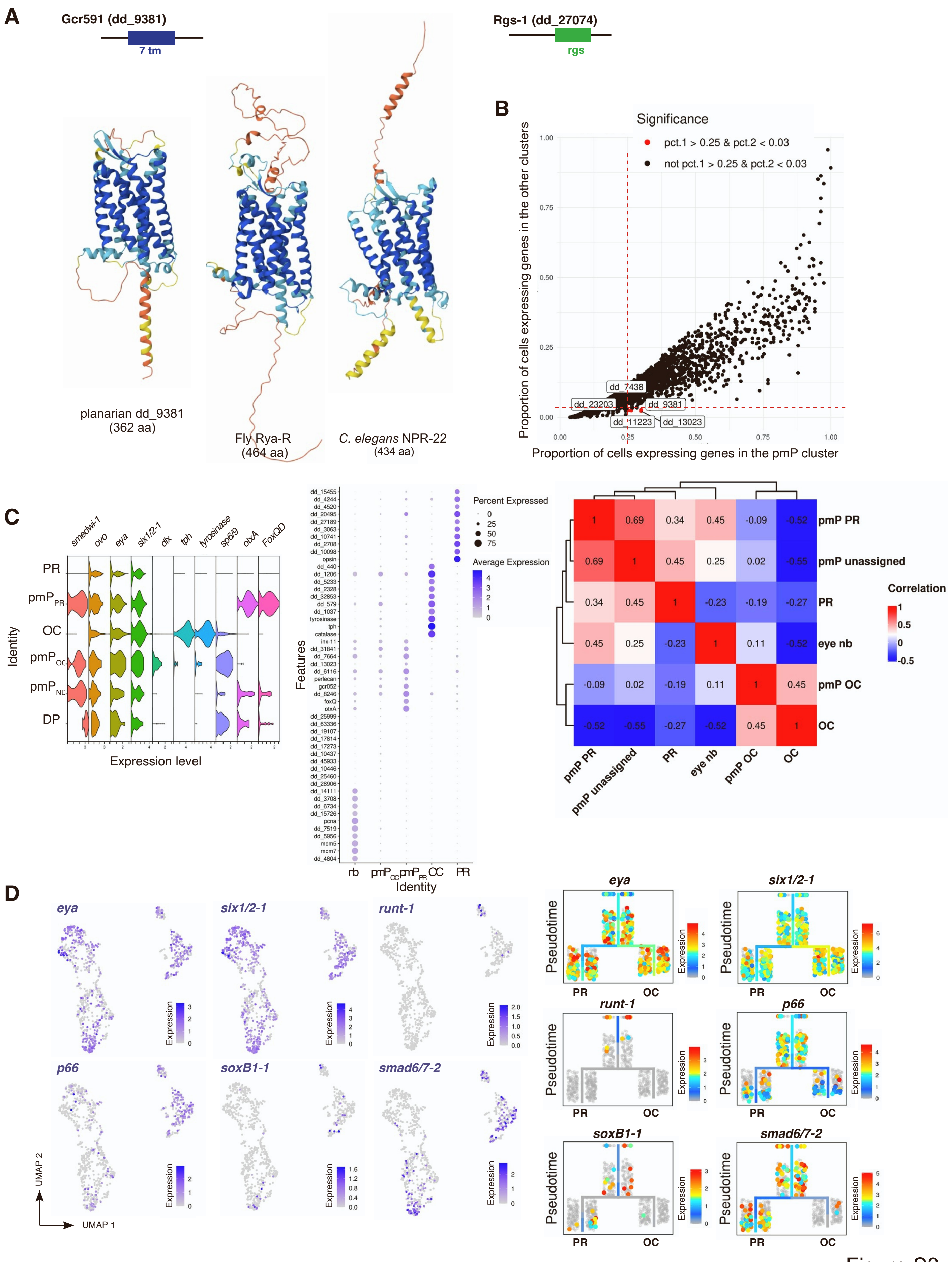
A
Gcr591 (dd_9381)
7 tm
Rgs-1 (dd_27074)
rgs
planarian dd_9381
(362 aa)
Fly Rya-R
(464 aa)
C. elegans NPR-22
(434 aa)
B
Significance
pct.1 > 0.25 & pct.2 < 0.03
not pct.1 > 0.25 & pct.2 < 0.03
Proportion of cells expressing genes in the other clusters
Proportion of cells expressing genes in the pmP cluster
dd_7438
dd_23203
dd_9381
dd_11223
dd_13023
C
smedwi-1
ovo
eya
six1/2-1
dlx
tph
tyrosinase
sp6/9
otxA
FoxQD
PR
pmP PR
OC
pmP OC
pmP ND
DP
Identity
Expression level
Features
Percent Expressed
Average Expression
Identity
Correlation
pmP PR
pmP unassigned
PR
eye nb
pmP OC
OC
D
eya
six1/2-1
runt-1
p66
soxB1-1
smad6/7-2
Expression
UMAP 1
UMAP 2
Pseudotime
PR
OC

Figure S3

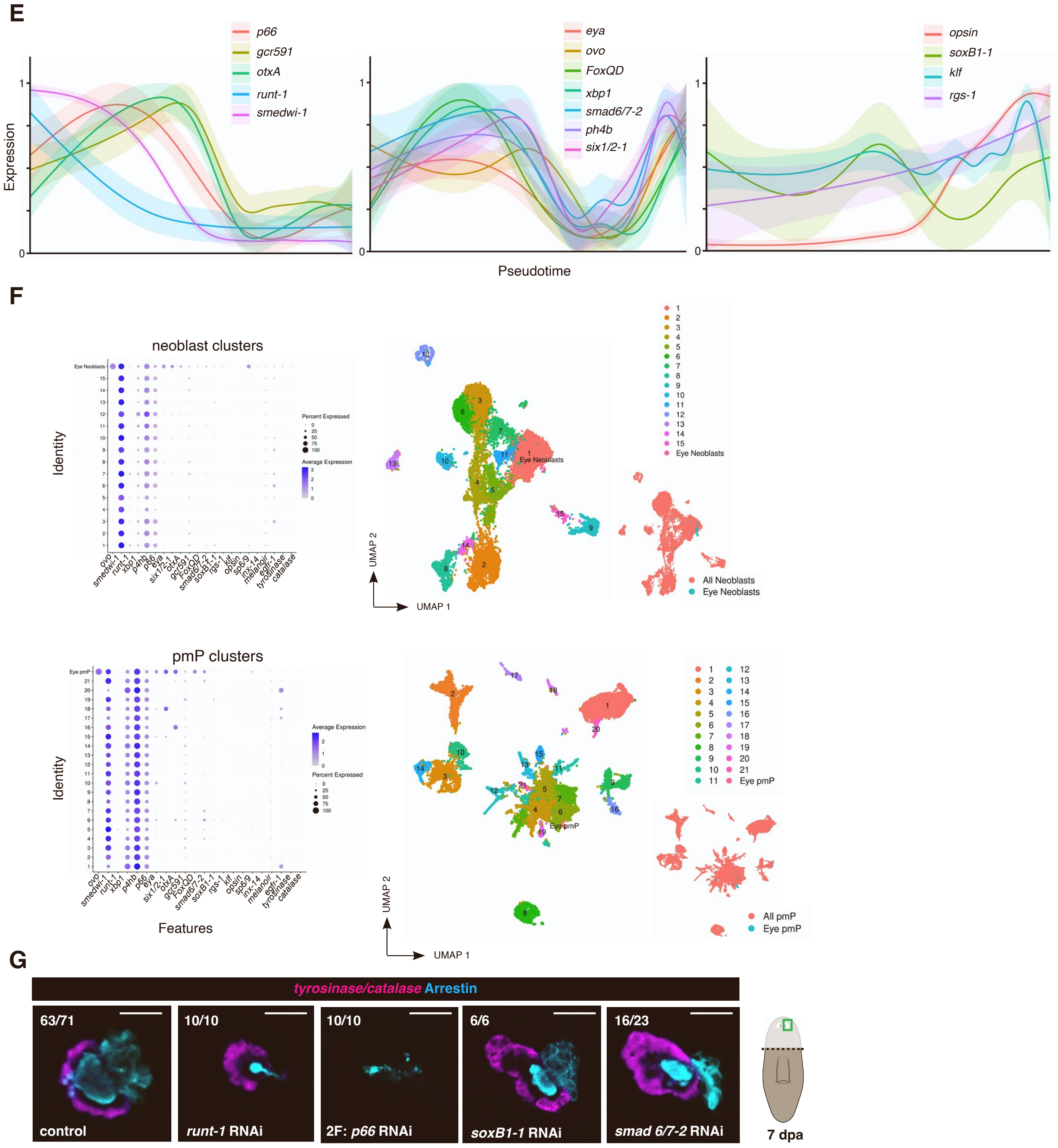
E
p66
gcr591
otxA
runt-1
smedwi-1
eya
ovo
FoxQD
xbp1
smad6/7-2
ph4b
six1/2-1
opsin
soxB1-1
klf
rgs-1
Expression
Pseudotime
F
neoblast clusters
Identity
Percent Expressed
Average Expression
All Neoblasts
Eye Neoblasts
UMAP 1
UMAP 2
pmP clusters
All pmP
Eye pmP
Features
G
tyrosinase/catalase Arrestin
63/71
control
10/10
runt-1 RNAi
10/10
2F: p66 RNAi
6/6
soxB1-1 RNAi
16/23
smad 6/7-2 RNAi
7 dpa

Figure S3

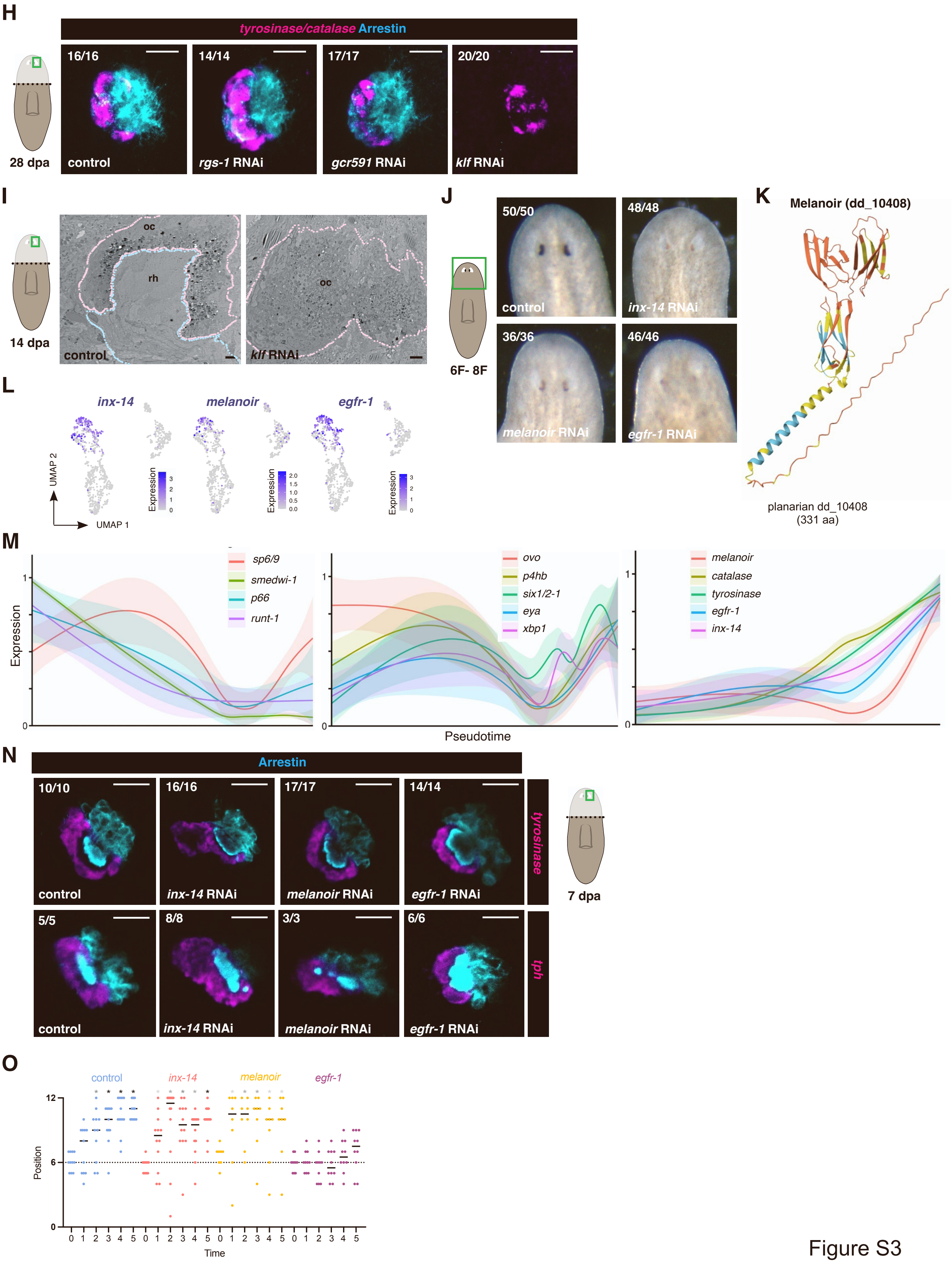
H
tyrosinase/catalase Arrestin
16/16
14/14
17/17
20/20
28 dpa
control
rgs-1 RNAi
gcr591 RNAi
klf RNAi
I
14 dpa
oc
rh
control
klf RNAi
J
50/50
48/48
36/36
46/46
6F- 8F
control
inx-14 RNAi
melanoir RNAi
egfr-1 RNAi
K
Melanoir (dd_10408)
planarian dd_10408
(331 aa)
L
inx-14
melanoir
egfr-1
UMAP 2
UMAP 1
Expression
M
Expression
sp6/9
smedwi-1
p66
runt-1
ovo
p4hb
six1/2-1
eya
xbp1
melanoir
catalase
tyrosinase
egfr-1
inx-14
Pseudotime
N
Arrestin
10/10
16/16
17/17
14/14
5/5
8/8
3/3
6/6
control
inx-14 RNAi
melanoir RNAi
egfr-1 RNAi
tyrosinase
tph
7 dpa
O
control
inx-14
melanoir
egfr-1
Position
Time

Figure S3

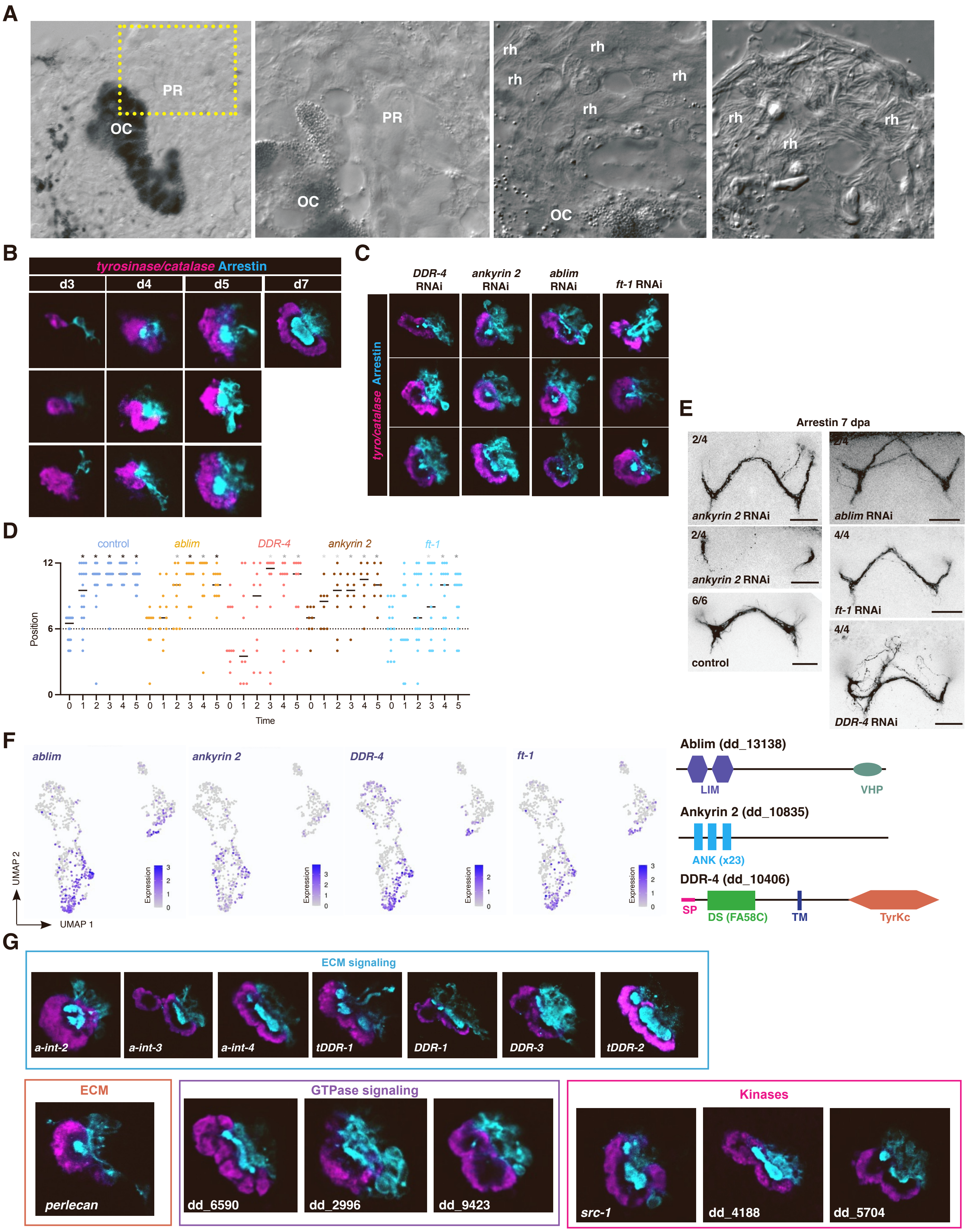
A
PR
OC
PR
OC
rh
rh
rh
rh
OC
rh
rh
rh
B
tyrosinase/catalase Arrestin
d3
d4
d5
d7
C
DDR-4 RNAi
ankyrin 2 RNAi
ablim RNAi
ft-1 RNAi
tyro/catalase Arrestin
D
control
ablim
DDR-4
ankyrin 2
ft-1
Position
Time
E
Arrestin 7 dpa
2/4
ankyrin 2 RNAi
2/4
ablim RNAi
2/4
ankyrin 2 RNAi
4/4
ft-1 RNAi
6/6
control
4/4
DDR-4 RNAi
F
ablim
ankyrin 2
DDR-4
ft-1
Expression
UMAP 2
UMAP 1
Ablim (dd_13138)
LIM
VHP
Ankyrin 2 (dd_10835)
ANK (x23)
DDR-4 (dd_10406)
SP
DS (FA58C)
TM
TyrKc
G
ECM signaling
a-int-2
a-int-3
a-int-4
tDDR-1
DDR-1
DDR-3
tDDR-2
ECM
perlecan
GTPase signaling
dd_6590
dd_2996
dd_9423
Kinases
src-1
dd_4188
dd_5704

Figure S4

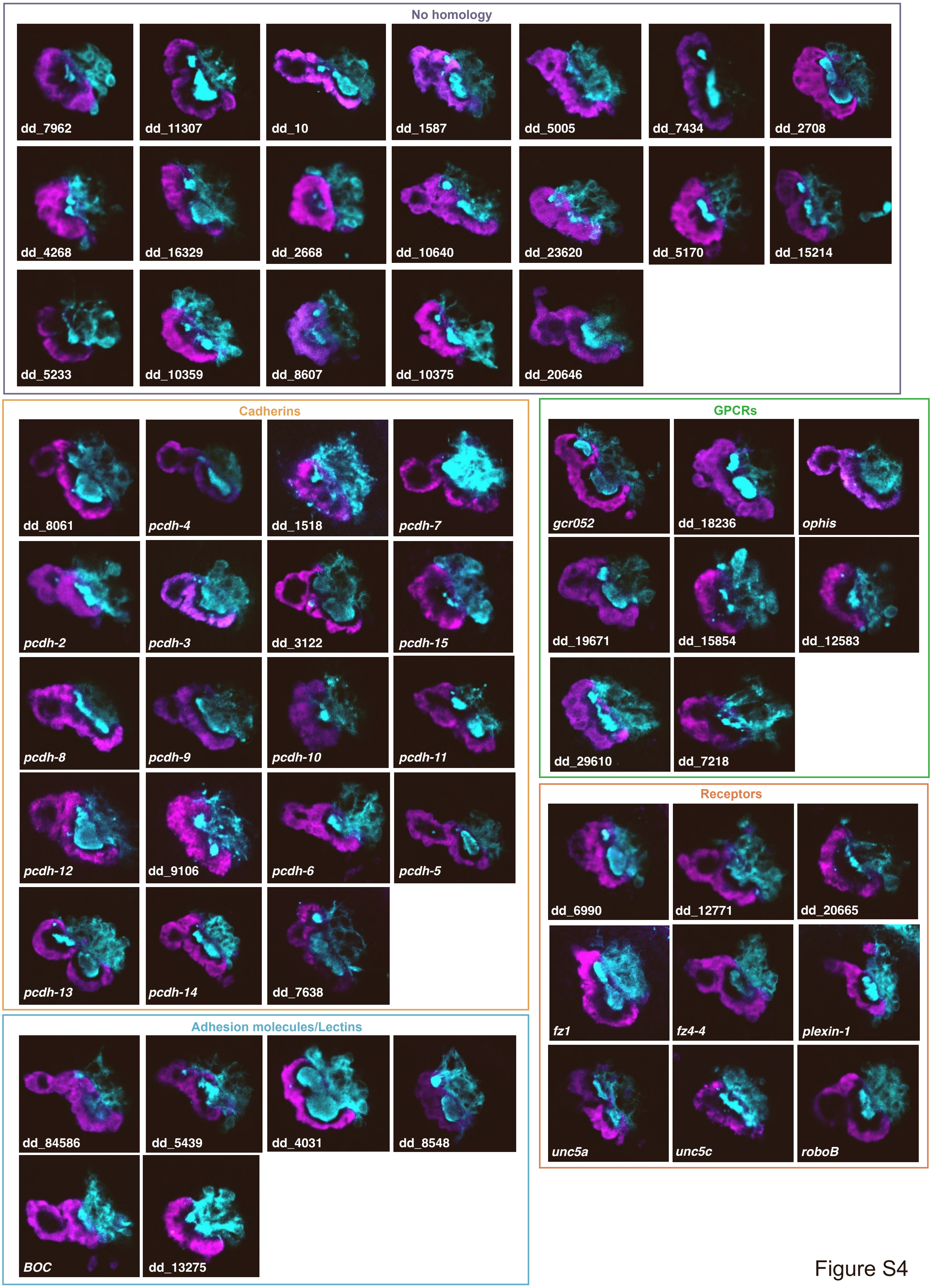
No homology
dd_7962
dd_11307
dd_10
dd_1587
dd_5005
dd_7434
dd_2708
dd_4268
dd_16329
dd_2668
dd_10640
dd_23620
dd_5170
dd_15214
dd_5233
dd_10359
dd_8607
dd_10375
dd_20646
Cadherins
dd_8061
pcdh-4
dd_1518
pcdh-7
pcdh-2
pcdh-3
dd_3122
pcdh-15
pcdh-8
pcdh-9
pcdh-10
pcdh-11
pcdh-12
dd_9106
pcdh-6
pcdh-5
pcdh-13
pcdh-14
dd_7638
GPCRs
gcr052
dd_18236
ophis
dd_19671
dd_15854
dd_12583
dd_29610
dd_7218
Receptors
dd_6990
dd_12771
dd_20665
fz1
fz4-4
plexin-1
unc5a
unc5c
roboB
Adhesion molecules/Lectins
dd_84586
dd_5439
dd_4031
dd_8548
BOC
dd_13275

Figure S4

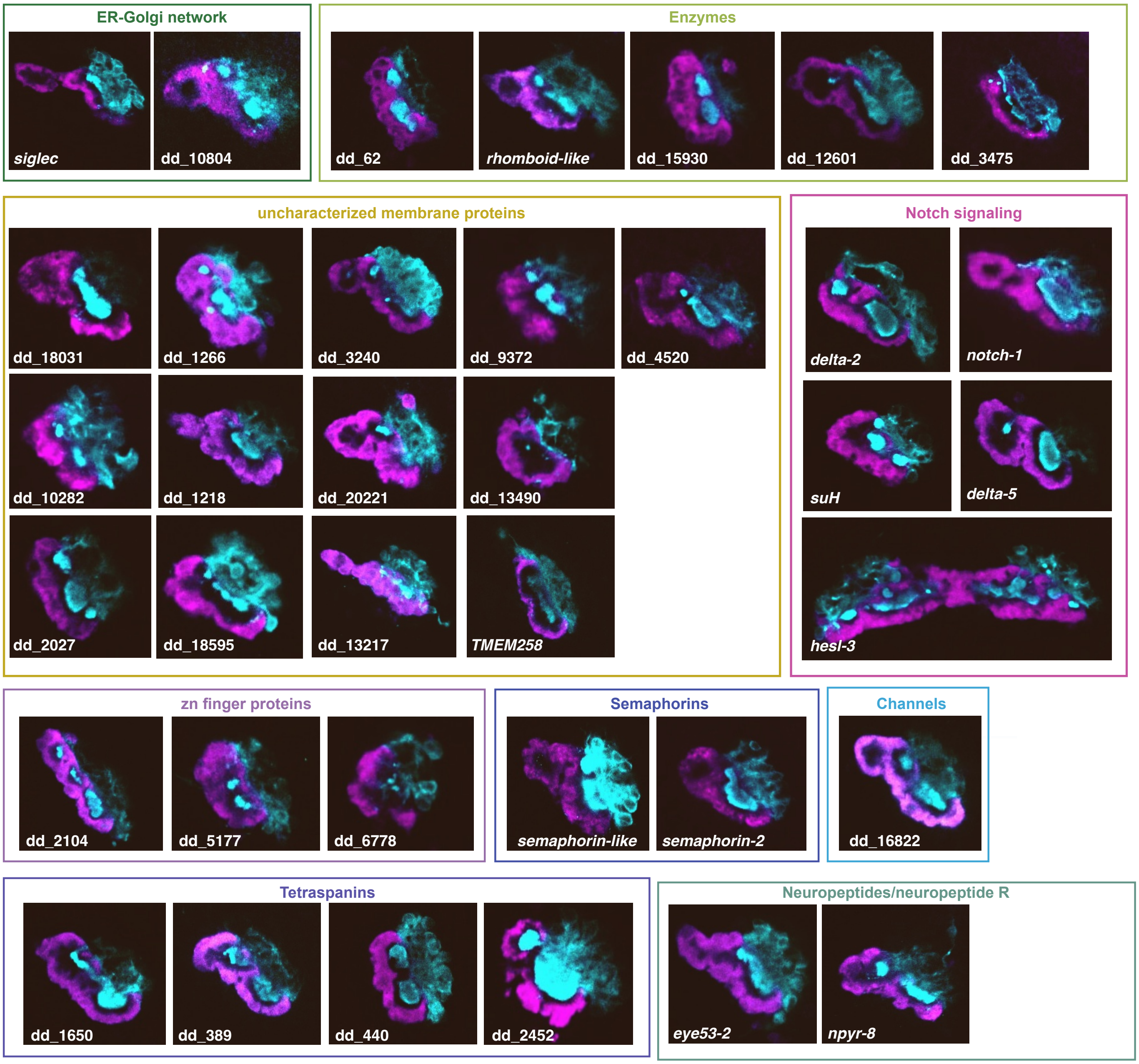
ER-Golgi network
siglec
dd_10804
Enzymes
dd_62
rhomboid-like
dd_15930
dd_12601
dd_3475
uncharacterized membrane proteins
dd_18031
dd_1266
dd_3240
dd_9372
dd_4520
dd_10282
dd_1218
dd_20221
dd_13490
dd_2027
dd_18595
dd_13217
TMEM258
Notch signaling
delta-2
notch-1
suH
delta-5
hesl-3
zn finger proteins
dd_2104
dd_5177
dd_6778
Semaphorins
semaphorin-like
semaphorin-2
Channels
dd_16822
Tetraspanins
dd_1650
dd_389
dd_440
dd_2452
Neuropeptides/neuropeptide R
eye53-2
npyr-8

H

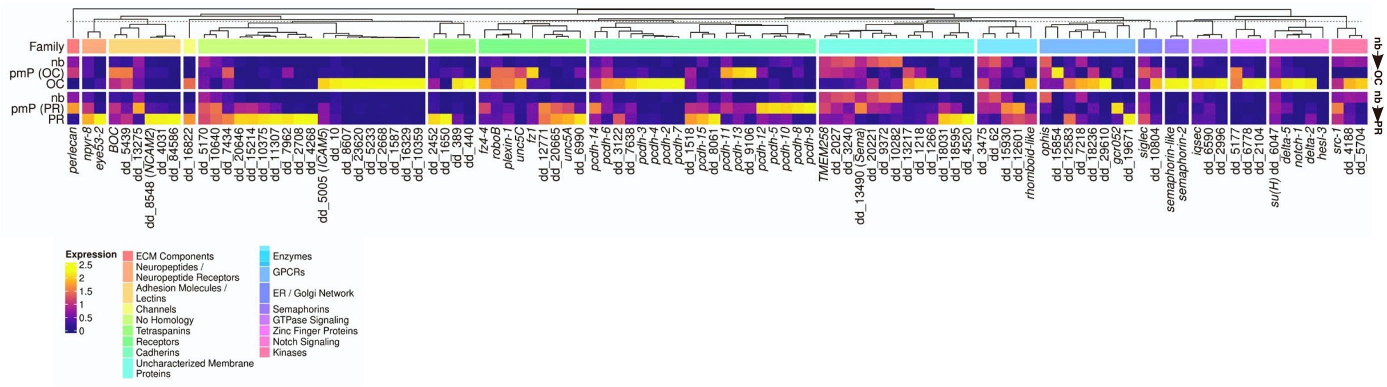
Family
nb
pmP (OC)
OC
nb
pmP (PR)
PR
Expression
2.5
2
1.5
1
0.5
0
ECM Components
Neuropeptides / Neuropeptide Receptors
Adhesion Molecules / Lectins
Channels
No Homology
Tetraspanins
Receptors
Cadherins
Uncharacterized Membrane Proteins
Enzymes
GPCRs
ER / Golgi Network
Semaphorins
GTPase Signaling
Zinc Finger Proteins
Notch Signaling
Kinases

Figure S4

I

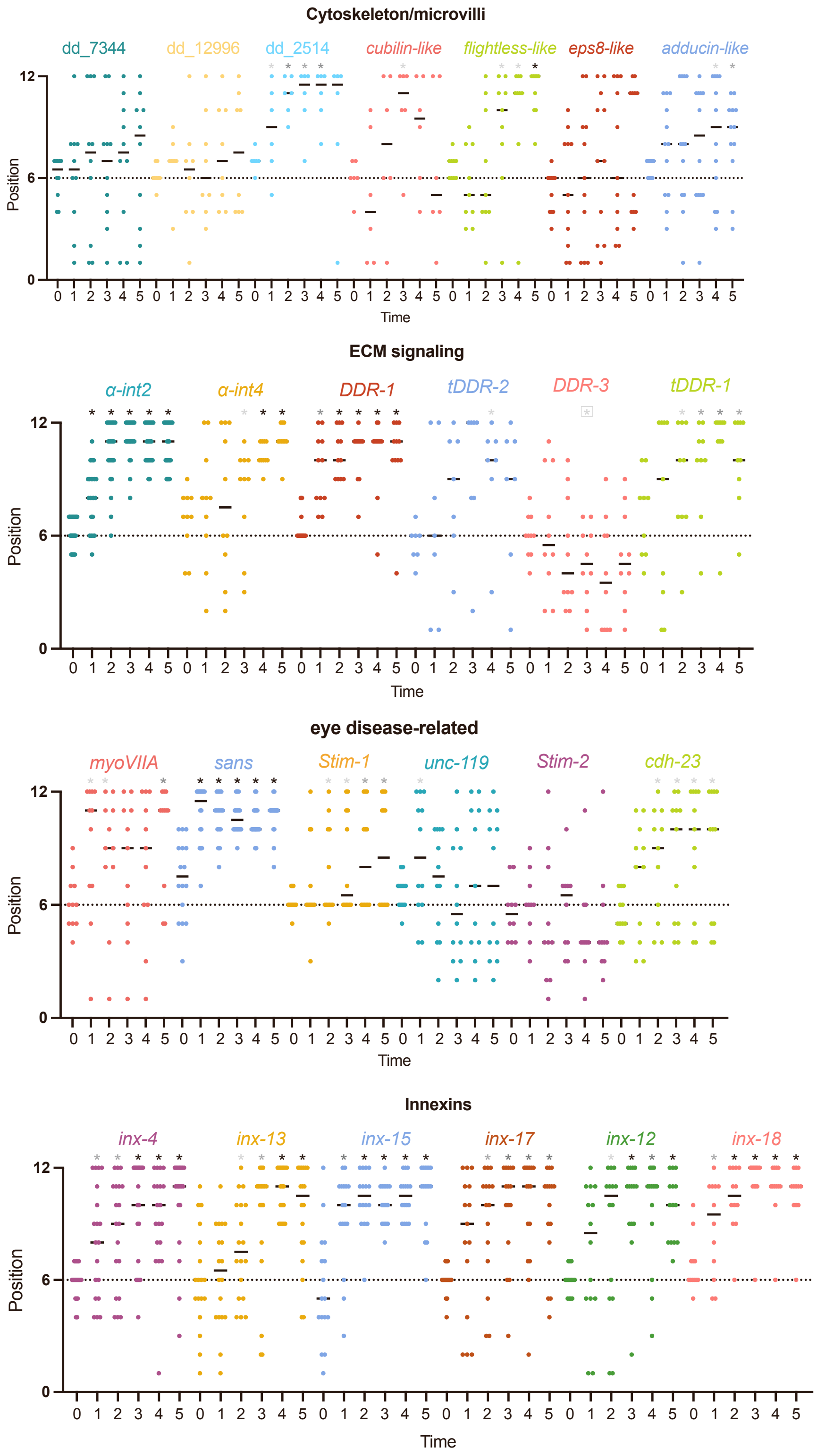
Cytoskeleton/microvilli
dd_7344
dd_12996
dd_2514
cubilin-like
flightless-like
eps8-like
adducin-like
Position
Time
ECM signaling
α-int2
α-int4
DDR-1
tDDR-2
DDR-3
tDDR-1
Position
Time
eye disease-related
myoVIIA
sans
Stim-1
unc-119
Stim-2
cdh-23
Position
Time
Innexins
inx-4
inx-13
inx-15
inx-17
inx-12
inx-18
Position
Time

Figure S4

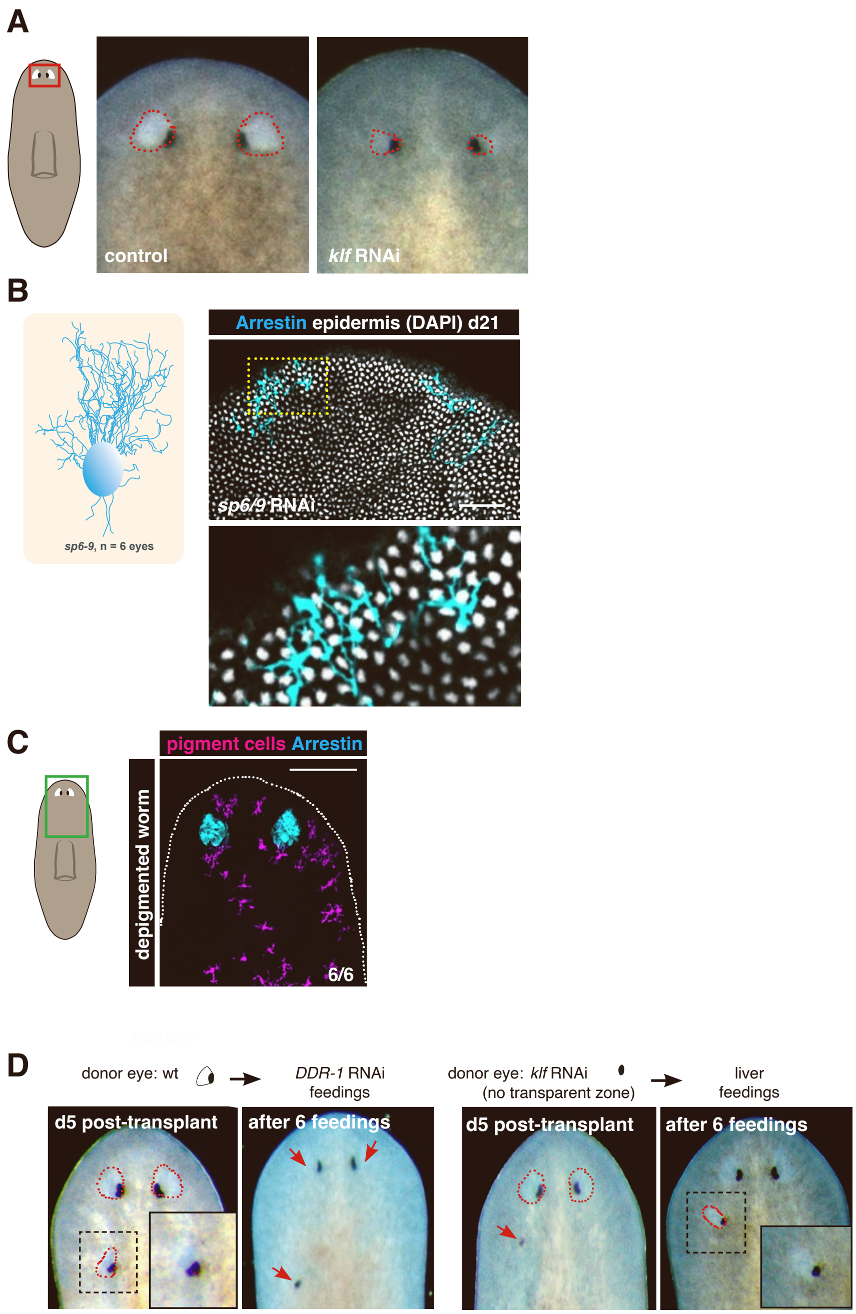
A
control
klf RNAi
B
Arrestin epidermis (DAPI) d21
sp6/9 RNAi
sp6-9, n = 6 eyes
C
pigment cells Arrestin
depigmented worm
6/6
D
donor eye: wt
DDR-1 RNAi feedings
donor eye: klf RNAi (no transparent zone)
liver feedings
d5 post-transplant
after 6 feedings
d5 post-transplant
after 6 feedings

Figure S5

E

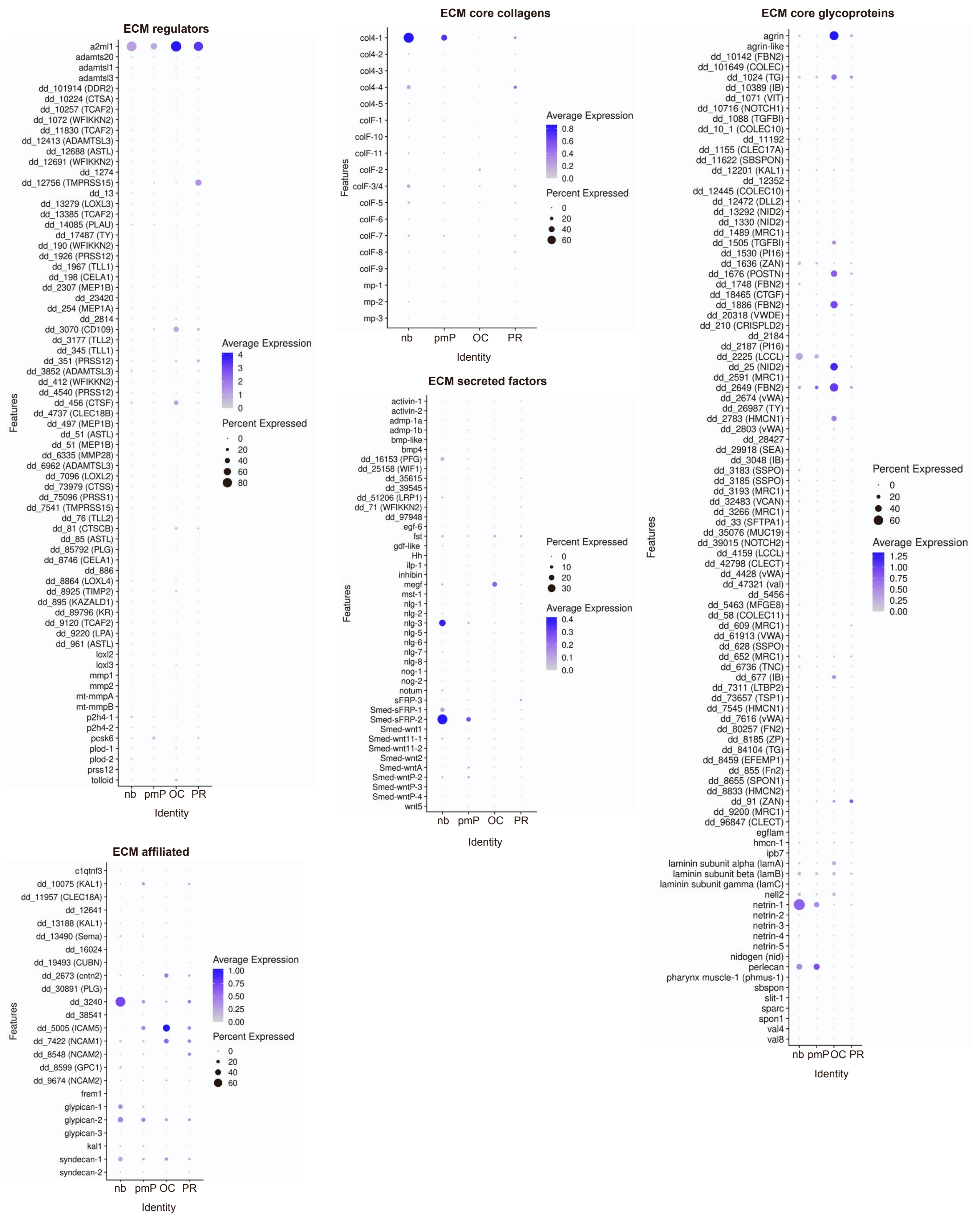
ECM regulators
a2ml1
adamts20
adamtsl1
adamtsl3
dd_101914 (DDR2)
dd_10224 (CTSA)
dd_10257 (TCAF2)
dd_1072 (WFIKKN2)
dd_11830 (TCAF2)
dd_12413 (ADAMTSL3)
dd_12688 (ASTL)
dd_12691 (WFIKKN2)
dd_1274
dd_12756 (TMPRSS15)
dd_13
dd_13279 (LOXL3)
dd_13385 (TCAF2)
dd_14085 (PLAU)
dd_17487 (TY)
dd_190 (WFIKKN2)
dd_1926 (PRSS12)
dd_1967 (TLL1)
dd_198 (CELA1)
dd_2307 (MEP1B)
dd_23420
dd_254 (MEP1A)
dd_2814
dd_3070 (CD109)
dd_3177 (TLL2)
dd_345 (TLL1)
dd_351 (PRSS12)
dd_3852 (ADAMTSL3)
dd_412 (WFIKKN2)
dd_4540 (PRSS12)
dd_456 (CTSF)
dd_4737 (CLEC18B)
dd_497 (MEP1B)
dd_51 (ASTL)
dd_51 (MEP1B)
dd_6335 (MMP28)
dd_6962 (ADAMTSL3)
dd_7096 (LOXL2)
dd_73979 (CTSS)
dd_75096 (PRSS1)
dd_7541 (TMPRSS15)
dd_76 (TLL2)
dd_81 (CTSCB)
dd_85 (ASTL)
dd_85792 (PLG)
dd_8746 (CELA1)
dd_886
dd_8864 (LOXL4)
dd_8925 (TIMP2)
dd_895 (KAZALD1)
dd_89796 (KR)
dd_9120 (TCAF2)
dd_9220 (LPA)
dd_961 (ASTL)
loxl2
loxl3
mmp1
mmp2
mt-mmpA
mt-mmpB
p2h4-1
p2h4-2
pcsk6
plod-1
plod-2
prss12
tolloid
Features
nb pmP OC PR
Identity
Average Expression
4
3
2
1
0
Percent Expressed
0
20
40
60
80
ECM core collagens
col4-1
col4-2
col4-3
col4-4
col4-5
colF-1
colF-10
colF-11
colF-2
colF-3/4
colF-5
colF-6
colF-7
colF-8
colF-9
mp-1
mp-2
mp-3
Features
nb pmP OC PR
Identity
Average Expression
0.8
0.6
0.4
0.2
0.0
Percent Expressed
0
20
40
60
ECM secreted factors
activin-1
activin-2
admp-1a
admp-1b
bmp-like
bmp4
dd_16153 (PFG)
dd_25158 (WIF1)
dd_35615
dd_39545
dd_51206 (LRP1)
dd_71 (WFIKKN2)
dd_97948
egf-6
fst
gdf-like
Hh
ilp-1
inhibin
megf
mst-1
nlg-1
nlg-2
nlg-3
nlg-5
nlg-6
nlg-7
nlg-8
nog-1
nog-2
notum
sFRP-3
Smed-sFRP-1
Smed-sFRP-2
Smed-wnt1
Smed-wnt11-1
Smed-wnt11-2
Smed-wnt2
Smed-wntA
Smed-wntP-2
Smed-wntP-3
Smed-wntP-4
wnt5
Features
nb pmP OC PR
Identity
Percent Expressed
0
10
20
30
Average Expression
0.4
0.3
0.2
0.1
0.0
ECM core glycoproteins
agrin
agrin-like
dd_10142 (FBN2)
dd_101649 (COLEC)
dd_1024 (TG)
dd_10389 (IB)
dd_1071 (VIT)
dd_10716 (NOTCH1)
dd_1088 (TGFBI)
dd_10_1 (COLEC10)
dd_11192
dd_1155 (CLEC17A)
dd_11622 (SBSPON)
dd_12201 (KAL1)
dd_12352
dd_12445 (COLEC10)
dd_12472 (DLL2)
dd_13292 (NID2)
dd_1330 (NID2)
dd_1489 (MRC1)
dd_1505 (TGFBI)
dd_1530 (PI16)
dd_1636 (ZAN)
dd_1676 (POSTN)
dd_1748 (FBN2)
dd_18465 (CTGF)
dd_1886 (FBN2)
dd_20318 (VWDE)
dd_210 (CRISPLD2)
dd_2184
dd_2187 (PI16)
dd_2225 (LCCL)
dd_25 (NID2)
dd_2591 (MRC1)
dd_2649 (FBN2)
dd_2674 (vWA)
dd_26987 (TY)
dd_2783 (HMCN1)
dd_2803 (vWA)
dd_28427
dd_29918 (SEA)
dd_3048 (IB)
dd_3183 (SSPO)
dd_3185 (SSPO)
dd_3193 (MRC1)
dd_32483 (VCAN)
dd_3266 (MRC1)
dd_33 (SFTPA1)
dd_35076 (MUC19)
dd_39015 (NOTCH2)
dd_4159 (LCCL)
dd_42798 (CLECT)
dd_4428 (vWA)
dd_47321 (val)
dd_5456
dd_5463 (MFGE8)
dd_58 (COLEC11)
dd_609 (MRC1)
dd_61913 (VWA)
dd_628 (SSPO)
dd_652 (MRC1)
dd_6736 (TNC)
dd_677 (IB)
dd_7311 (LTBP2)
dd_73657 (TSP1)
dd_7545 (HMCN1)
dd_7616 (vWA)
dd_80257 (FN2)
dd_8185 (ZP)
dd_84104 (TG)
dd_8459 (EFEMP1)
dd_855 (Fn2)
dd_8655 (SPON1)
dd_8833 (HMCN2)
dd_91 (ZAN)
dd_9200 (MRC1)
dd_96847 (CLECT)
egflam
hmcn-1
ipb7
laminin subunit alpha (lamA)
laminin subunit beta (lamB)
laminin subunit gamma (lamC)
nell2
netrin-1
netrin-2
netrin-3
netrin-4
netrin-5
nidogen (nid)
perlecan
pharynx muscle-1 (phmus-1)
sbspon
slit-1
sparc
spon1
val4
val8
Features
nb pmP OC PR
Identity
Percent Expressed
0
20
40
60
Average Expression
1.25
1.00
0.75
0.50
0.25
0.00
ECM affiliated
c1qtnf3
dd_10075 (KAL1)
dd_11957 (CLEC18A)
dd_12641
dd_13188 (KAL1)
dd_13490 (Sema)
dd_16024
dd_19493 (CUBN)
dd_2673 (cntn2)
dd_30891 (PLG)
dd_3240
dd_38541
dd_5005 (ICAM5)
dd_7422 (NCAM1)
dd_8548 (NCAM2)
dd_8599 (GPC1)
dd_9674 (NCAM2)
frem1
glypican-1
glypican-2
glypican-3
kal1
syndecan-1
syndecan-2
Features
nb pmP OC PR
Identity
Average Expression
1.00
0.75
0.50
0.25
0.00
Percent Expressed
0
20
40
60

Figure S5

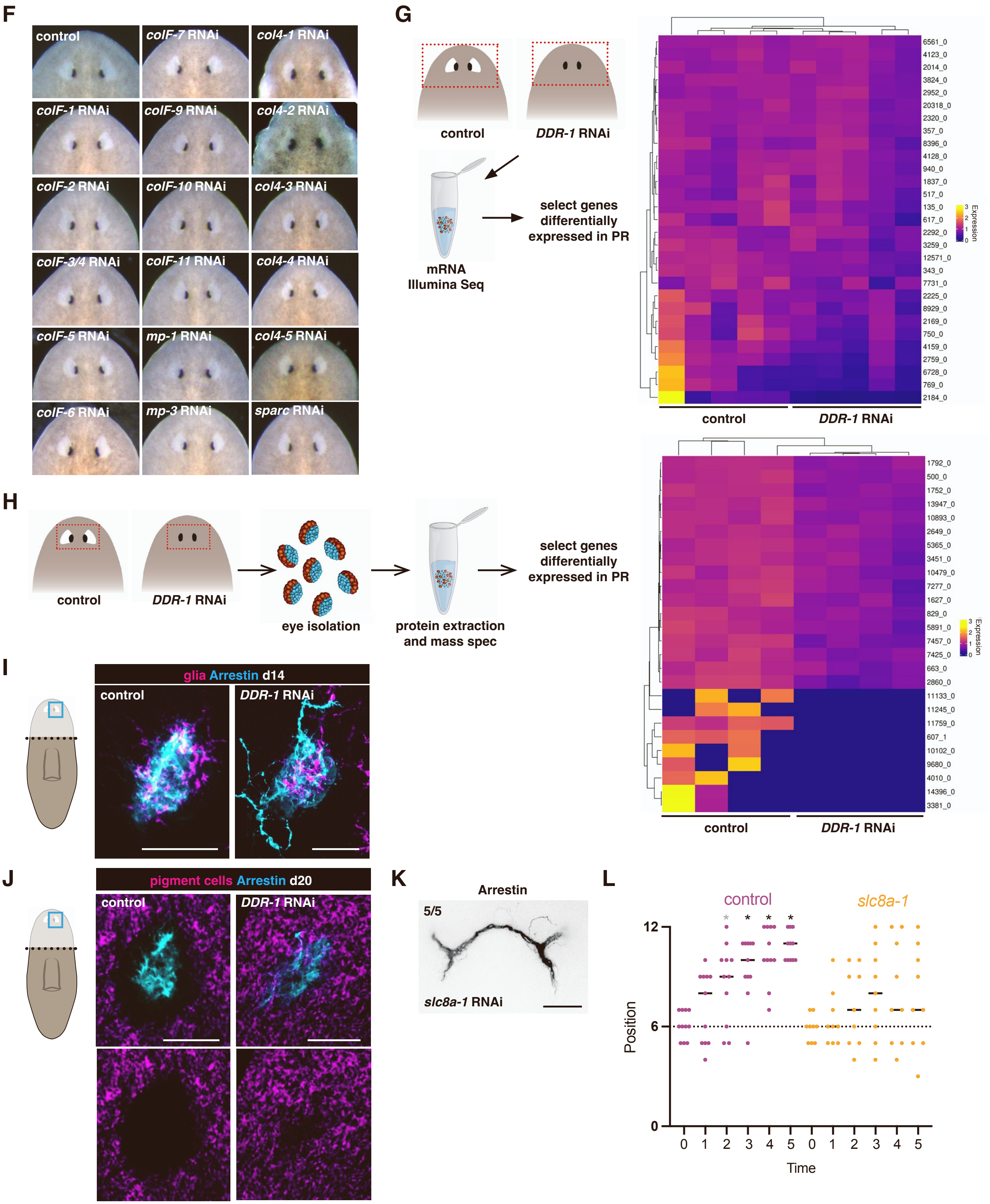
F
control
colF-7 RNAi
col4-1 RNAi
colF-1 RNAi
colF-9 RNAi
col4-2 RNAi
colF-2 RNAi
colF-10 RNAi
col4-3 RNAi
colF-3/4 RNAi
colF-11 RNAi
col4-4 RNAi
colF-5 RNAi
mp-1 RNAi
col4-5 RNAi
colF-6 RNAi
mp-3 RNAi
sparc RNAi
G
control
DDR-1 RNAi
mRNA
Illumina Seq
select genes differentially expressed in PR
6561_0
4123_0
2014_0
3824_0
2952_0
20318_0
2320_0
357_0
8396_0
4128_0
940_0
1837_0
517_0
135_0
617_0
2292_0
3259_0
12571_0
343_0
7731_0
2225_0
8929_0
2169_0
750_0
4159_0
2759_0
6728_0
769_0
2184_0
Expression
control
DDR-1 RNAi
1792_0
500_0
1752_0
13947_0
10893_0
2649_0
5365_0
3451_0
10479_0
7277_0
1627_0
829_0
5891_0
7457_0
7425_0
663_0
2860_0
11133_0
11245_0
11759_0
607_1
10102_0
9680_0
4010_0
14396_0
3381_0
Expression
control
DDR-1 RNAi
H
control
DDR-1 RNAi
eye isolation
protein extraction and mass spec
select genes differentially expressed in PR
I
glia Arrestin d14
control
DDR-1 RNAi
J
pigment cells Arrestin d20
control
DDR-1 RNAi
K
Arrestin
5/5
slc8a-1 RNAi
L
control
slc8a-1
12
6
0
Position
0 1 2 3 4 5 0 1 2 3 4 5
Time

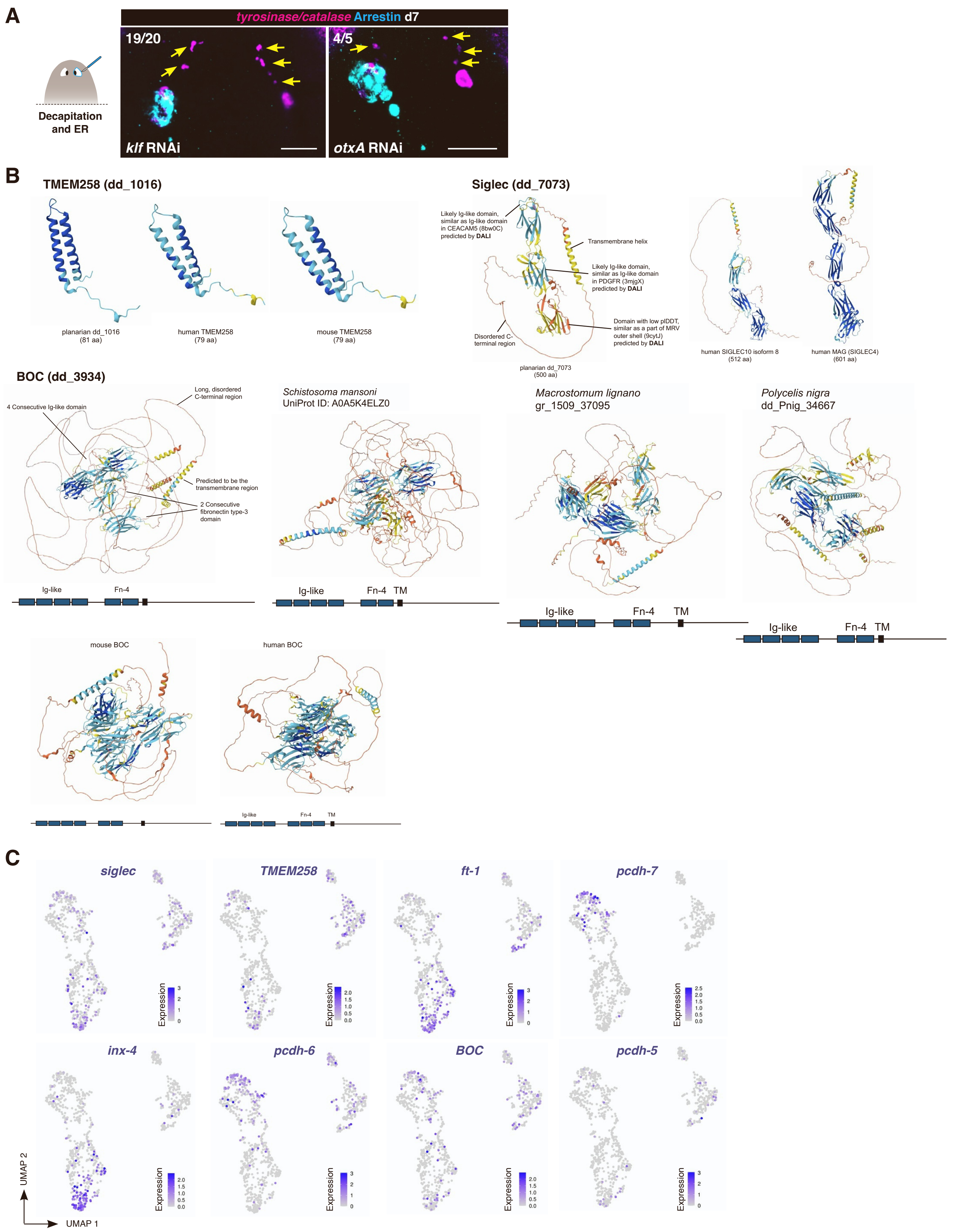
A
tyrosinase/catalase Arrestin d7
Decapitation and ER
19/20
4/5
klf RNAi
otxA RNAi
B
TMEM258 (dd_1016)
planarian dd_1016 (81 aa)
human TMEM258 (79 aa)
mouse TMEM258 (79 aa)
Siglec (dd_7073)
Likely Ig-like domain, similar as Ig-like domain in CEACAM5 (8bw0C) predicted by DALI
Transmembrane helix
Likely Ig-like domain, similar as Ig-like domain in PDGFR (3mjgX) predicted by DALI
Domain with low pIDDT, similar as a part of MRV outer shell (9cytJ) predicted by DALI
Disordered C-terminal region
planarian dd_7073 (500 aa)
human SIGLEC10 isoform 8 (512 aa)
human MAG (SIGLEC4) (601 aa)
BOC (dd_3934)
Long, disordered C-terminal region
4 Consecutive Ig-like domain
Predicted to be the transmembrane region
2 Consecutive fibronectin type-3 domain
Ig-like
Fn-4
Schistosoma mansoni UniProt ID: A0A5K4ELZ0
Macrostomum lignano gr_1509_37095
Polycelis nigra dd_Pnig_34667
TM
mouse BOC
human BOC
C
siglec
TMEM258
ft-1
pcdh-7
inx-4
pcdh-6
BOC
pcdh-5
Expression
UMAP 1
UMAP 2

Figure S6

D

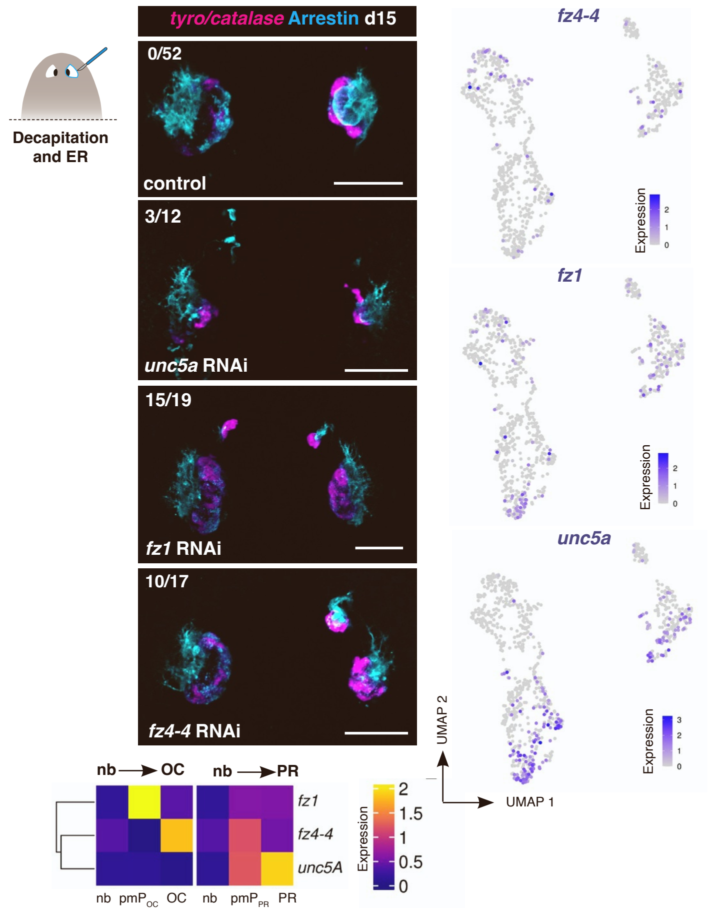

Decapitation and ER
tyro/catalase Arrestin d15
0/52
control
3/12
unc5a RNAi
15/19
fz1 RNAi
10/17
fz4-4 RNAi
fz4-4
fz1
unc5a
Expression
UMAP 2
UMAP 1
nb → OC
nb → PR
fz1
fz4-4
unc5A
nb
pmP_OC
OC
nb
pmP_PR
PR
Expression
2
1.5
1
0.5
0

E

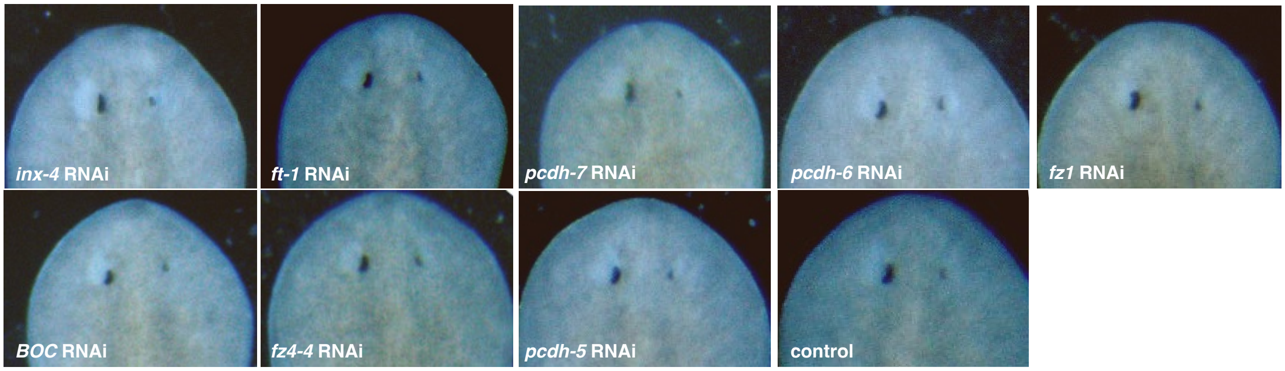

inx-4 RNAi
ft-1 RNAi
pcdh-7 RNAi
pcdh-6 RNAi
fz1 RNAi
BOC RNAi
fz4-4 RNAi
pcdh-5 RNAi
control

Figure S6

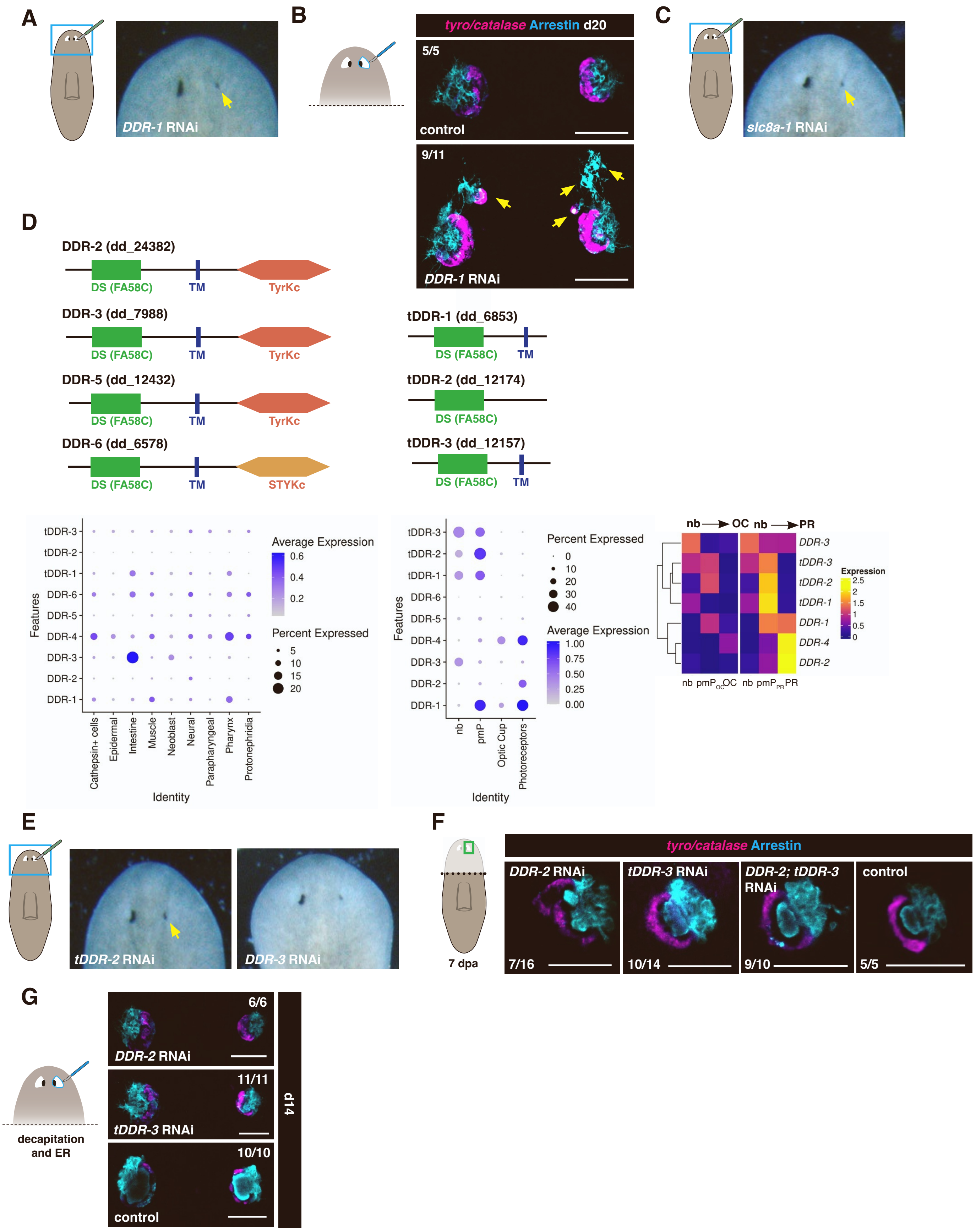
A
DDR-1 RNAi
B
tyro/catalase Arrestin d20
5/5
control
9/11
DDR-1 RNAi
C
slc8a-1 RNAi
D
DDR-2 (dd_24382)
DS (FA58C)
TM
TyrKc
DDR-3 (dd_7988)
DDR-5 (dd_12432)
DDR-6 (dd_6578)
STYKc
tDDR-1 (dd_6853)
tDDR-2 (dd_12174)
tDDR-3 (dd_12157)
Features
Average Expression
Percent Expressed
Identity
Cathepsin+ cells
Epidermal
Intestine
Muscle
Neoblast
Neural
Parapharyngeal
Pharynx
Protonephridia
nb
pmP
Optic Cup
Photoreceptors
nb → OC nb → PR
Expression
E
tDDR-2 RNAi
DDR-3 RNAi
F
tyro/catalase Arrestin
DDR-2 RNAi
tDDR-3 RNAi
DDR-2; tDDR-3 RNAi
control
7 dpa
7/16
10/14
9/10
5/5
G
6/6
DDR-2 RNAi
11/11
tDDR-3 RNAi
10/10
control
d14
decapitation and ER

Figure S7
